# Sulforaphane decreases serum selenoprotein P levels through enhancement of lysosomal degradation independent of Nrf2

**DOI:** 10.1038/s42003-023-05449-y

**Published:** 2023-10-19

**Authors:** Xinying Ye, Takashi Toyama, Keiko Taguchi, Kotoko Arisawa, Takayuki Kaneko, Ryouhei Tsutsumi, Masayuki Yamamoto, Yoshiro Saito

**Affiliations:** 1 Laboratory of Molecular Biology and Metabolism, Graduate School of Pharmaceutical Sciences, Tohoku University, 6-3 Aoba, Aramaki, Aoba-ku, Sendai, Miyagi 980-8578 Japan; 2 Department of Medical Biochemistry, Tohoku University Graduate School of Medicine, 2-1 Seiryo-machi, Aoba-ku, Sendai, Miyagi 980-8575 Japan

**Keywords:** Molecular medicine, Lysosomes, Stress signalling

## Abstract

Selenoprotein P (SeP) is a major selenoprotein in serum predominantly produced in the liver. Excess SeP impairs insulin secretion from the pancreas and insulin sensitivity in skeletal muscle, thus inhibition of SeP could be a therapeutic strategy for type 2 diabetes. In this study, we examine the effect of sulforaphane (SFN), a phytochemical of broccoli sprouts and an Nrf2 activator, on SeP expression in vitro and in vivo. Treatment of HepG2 cells with SFN decreases inter- and intra-cellular SeP levels. SFN enhances lysosomal acidification and expression of V-ATPase, and inhibition of this process cancels the decrease of SeP by SFN. SFN activates Nrf2 in the cells, while Nrf2 siRNA does not affect the decrease of SeP by SFN or lysosomal acidification. These results indicate that SFN decreases SeP by enhancing lysosomal degradation, independent of Nrf2. Injection of SFN to mice results in induction of cathepsin and a decrease of SeP in serum. The findings from this study are expected to contribute to developing SeP inhibitors in the future, thereby contributing to treating and preventing diseases related to increased SeP.

## Abstract (web summary)

Sulforaphane, a plant secondary metabolite found in crucifers, decreases selenoprotein P levels by enhancing lysosomal degradation, suggesting that this phytochemical is a candidate for controlling selenoprotein P-associated diseases like type 2 diabetes.

## Introduction

Selenium is an essential trace element and has recently become a potential target for cancer chemotherapy. However, the Selenium and Vitamin E Cancer Prevention Trial (SELECT), an intervention study for selenium supplementation conducted on 29,133 people in the United States, found that supplementation of selenium was ineffective in preventing cancer and could instead increase diabetic risk^1^. In another intervention study conducted on 1312 people, the National Prevention of Cancer (NPC), found that selenium supplementation significantly increased the risk of type 2 diabetes^2^. Therefore, excessive selenium intake is a risk factor for diabetes.

Selenium undergoes various changes in the cell and is incorporated into proteins such as selenocysteine, a rare amino acid in which the sulfur atom of cysteine is replaced with selenium. The human genome encodes 25 types of selenocysteine-containing proteins, also known as selenoproteins, which include important reductases such as thioredoxin reductase (TrxR) and glutathione peroxidase (GPx)^3^. These enzymes possess selenocysteine as their catalytic center and are essential for the maintenance of cellular redox status, thus, an appropriate concentration of selenium is necessary for life. Selenoprotein P (SeP; encoded by *Selenop*) is an extremely unique selenoprotein that contains 10 selenocysteines per molecule^4^, and is mainly synthesized in the liver and then secreted into plasma. The majority (53%) of the selenium present in plasma is contained in SeP^5^. Extracellular SeP can bind to apolipoprotein E receptor 2 (ApoER2), an LDL receptor, and be taken up by cells via endocytosis^6,7^. Incorporated SeP is readily degraded by lysosomes and acts as a source of selenium, supplying selenocysteine to cells^8^. In other words, SeP is responsible for supplying selenium from the liver to the entire body. We recently found that excessive SeP production is associated with type 2 diabetes^9,10^. Epidemiological studies revealed that plasma SeP levels increased in type 2 diabetes, and this increase has a significant correlation with insulin resistance^11,12^. We also clarified that SeP causes insulin resistance by inhibition of AMP-activated protein kinase (AMPK) in cultured hepatoma and myotube cells^7,13^. Furthermore, it was hypothesized that SeP suppresses insulin secretion by damaging pancreatic β cells. Administration of a neutralizing antibody against SeP to type 2 diabetes model KKAy mouse improved insulin sensitivity and glucose tolerance, suggesting that SeP is a biomarker of diabetes and a possible therapeutic target^10^.

Though the regulatory mechanism of SeP expression in the liver has not been completely clarified, it is known that transcription factors such as SREBP-1, HIF-1α, and FOXO1 are involved in increasing its expression^13–15^. Recently, we also found that CCDC152 (L-IST), a long non-coding RNA with a sequence complementary to the selenocysteine insertion sequence (SECIS) of SeP, is induced by epigallocatechin gallate and suppresses the translation of SeP^16^. Therefore, we thought that functional phytochemicals would be useful in lowering SeP expression. A recent study suggested that sulforaphane (SFN), a phytochemical derived from cruciferous plants, inhibits SREBP-1, hence we hypothesized that SFN could be a candidate for SeP suppression and relieve diabetic symptoms^17^. Normally, SFN exists as a stable glycoside in cruciferous plants such as broccoli, brussels sprouts, and cabbage. When the plant is damaged, the glycoside is desorbed by myrosinase, generating SFN^18,19^. SFN has an isothiocyanate group (R-N=C=S) that covalently binds with the pain receptor, TRPA1, on the tongue, resulting in a pungent taste. Therefore, this molecule is thought to contribute to the self-defense of cruciferous plants^20^. It is also known that even when ingested as a glycoside, it is eventually converted to SFN by intestinal bacteria^21^. In addition, SFN has been found to be a useful phytochemical for cancer prevention from research at Johns Hopkins University and has attracted attention as a dietary supplement. It has been reported that one of the underlying molecular mechanisms of SFN is the activation of the Nrf2 pathway, which is a master regulator of phase 2 detoxifying enzymes^22^. Interestingly, administration of SFN to diabetic model high fat diet-fed mice have been reported to improve weight gain and pathology^23^, however its molecular mechanisms are not well understood. Taken together, this study aims to examine the effects of SFN on SeP production and to elucidate its regulatory mechanisms. The results of the present study demonstrate that SFN suppresses SeP production from the liver via an Nrf2-independent mechanism.

## Results

### Suppressive effects of SFN on selenite-induced SeP expression in HepG2 cells

**Fig. 1:**
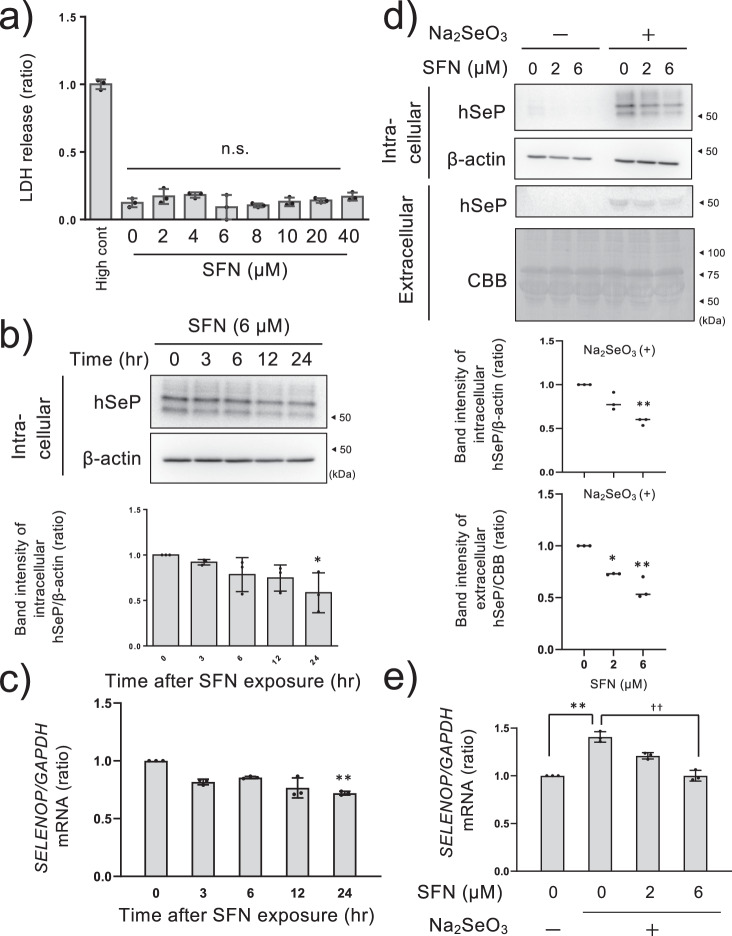
Suppressive effects of SFN on SeP at the protein and mRNA levels in HepG2 cells. HepG2 cells were cultured in high glucose DMEM containing selenite (100 nM) for 24 h and treated with SFN at the indicated concentration for 24 h. The cytotoxicity was measured by LDH assay (**a**). The data is expressed as Mean ± S.D. (*n* = 3), and is shown as a relative value with the lysed control (High cont) as 1. HepG2 cells were cultured in high glucose DMEM containing selenite (100 nM) for 24 h and treated with 6 µM SFN for the indicated time. Then cell lysate was collected and SeP was detected by western blotting (upper panel) and quantified (lower panel. *n* = 3) (**b**). The total RNA was collected and *SELENOP* mRNA was detected by RT-qPCR (**c**). The data is expressed as Mean ± S.D (*n* = 3). The cells were treated with SFN at the indicated concentration for 24 h. Then the culture medium and cell lysate were collected and SeP was detected by western blotting (upper panel) and quantified (lower panel, *n* = 3) (**d**). Total RNA was collected and *SELENOP* mRNA was detected by RT-qPCR (**e**). Intracellular and extracellular SeP were corrected with β-actin or CBB-stained total proteins respectively. Quantitative data is shown as a relative value with the control as 1. *P < 0.05 and **P < 0.01 vs. control. One-way ANOVA, post hoc test Dunnett method were used for statistical analysis. All blots were performed on independent membranes and were done with the same sample volume applied. CBB staining was performed on the same membrane.

It has been reported that HepG2 cells produce SeP and release it into the medium at maximal intensity in the presence of 64–128 nM of selenium^24^. We used a human hepatoma HepG2 cell line, as a model cell that expresses SeP and secretes it into the medium, and cultured the cells in a high glucose medium supplemented with a trace amount of selenite (100 nM)^7,24^. Since SFN is electrophilic, it may cause cytotoxicity via covalent modifications of intracellular proteins^25^. The damaging effects of SFN against Helicobacter pylori and cancer cells may be due to its electrophilicity^26^. Therefore, we first investigated the toxicity of SFN against HepG2 cells. Cell death was determined by the amount of lactate dehydrogenase (LDH) released. Significant cell death was not observed when the cells were treated with up to 40 µM SFN for 24 h (Fig. 1a). We also evaluated the effect of SNF on SeP at the protein levels in a concentration-dependent manner and found that the decrease of SeP reached a plateau at 6 µM (Supplementary Fig. S1). In the present study, 6 µM SFN was used as the maximum concentration that did not exhibit cellular toxicity.

To examine whether SFN reduced SeP at the protein and mRNA level in hepatocytes, HepG2 cells cultured in high glucose medium supplemented with 100 nM selenite were treated with 6 µM SFN for the indicated time course. As a result, intracellular SeP at the protein level significantly decreased after 24 h treatment of SFN (Fig. 1b). Under the same conditions, *SELENOP* mRNA levels were also partially decreased by SFN after 24 h (Fig. 1c). The cells were then treated with SFN at 2 and 6 µM for 24 h and the intra- and extracellular SeP protein levels in the absence and presence of 100 nM selenite were examined. As the band position of extracellular SeP was disturbed by excess albumin (66 kDa), the observed band patterns appeared to be different from those of intracellular SeP (Fig. 1d). SeP undergoes glycosylation, thus SeP was detected at multiple band patterns (Supplementary Fig. S2 indicates that SeP glycosylation was the cause of the variance in molecular weight). We also checked that all bands were diminished by knocking out SeP in HepG2 cells, thus we quantified SeP as a sum of multiple bands around 60 kDa. In the absence of selenite, the amount of SeP detected was negligible. As well, selenite-induced inter- and intracellular SeP production was lowered by SFN in a dose-dependent manner (Fig. 1d). Selenite-induced *SELENOP* mRNA was also decreased by SFN under these conditions (Fig. 1e). These results suggest that SFN suppresses SeP production via inhibition of its transcription and its subsequent extracellular release. However, the decrease of *SELENOP* mRNA level by SFN was partial and did not correlate with the decrease of SeP at the protein level. Treatment with SFN at the maximum concentration (6 µM) lowered *SELENOP* mRNA expression to around the same as the untreated control, however, protein expression was maintained under the same conditions. Thus, we further evaluated the implications of SeP protein degradation.

### Contributions of lysosome to the downregulation of SeP by SFN

**Fig. 2:**
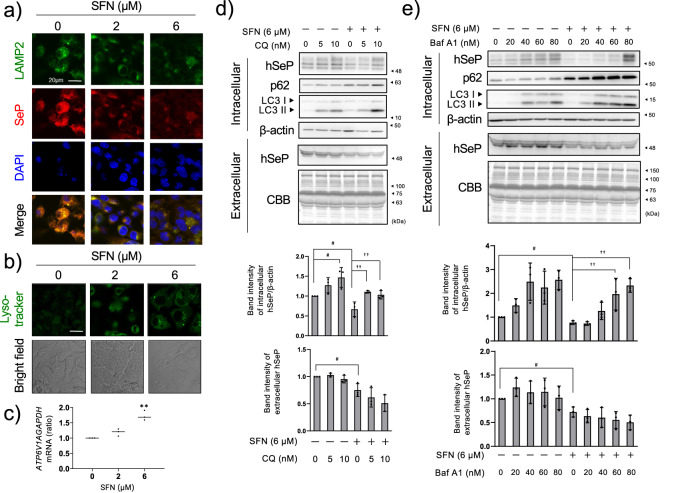
Involvement of lysosomes in the reduction of SeP by SFN. HepG2 cells were cultured in high glucose DMEM containing selenite (100 nM) for 24 h and treated with SFN at the indicated concentration for 24 h. Then, immunostaining was performed using LAMP2 and SeP antibodies. Fluorescence was observed with a confocal microscope (**a**). The scale bar indicates 20 µm. HepG2 cells were cultured in high glucose DMEM containing selenite (100 nM) for 24 h and treated with SFN at the indicated concentration for 24 h. After that, the cells were treated with Lysotracker, and fluorescence was observed with a confocal microscope (**b**). The scale bar indicates 20 µm. The cells were treated with SFN at the indicated concentration for 24 h. *ATP6V1A* mRNA was measured using RT-qPCR (**c**). HepG2 cells were cultured in high glucose DMEM containing selenite (100 nM) for 24 h and treated with 6 µM SFN in the presence of the indicated concentration of chloroquine (CQ) (**d**) or bafilomycin A1 (BafA1) (**e**) for 24 h. The culture medium supernatant and cell lysate were collected, and western blotting was performed. Quantitative data is shown at the lower panel of each figure and indicated as a relative value with the control as 1. The data is expressed as mean ± S.D. (*n* = 3). *P < 0.05 and **P < 0.01 vs. control. One-way ANOVA, post hoc test Dunnett method were used for statistical analysis. All blots were performed on independent membranes and were done with the same sample volume applied. CBB staining was performed on the same membrane.

Since extracellular SeP is taken up by endocytosis and degraded in the lysosome, and because some groups have reported that SFN activates lysosomal function, we hypothesized that SFN may enhance SeP degradation through activation of the lysosome^8,27^. Immunocytochemical analyses indicated that selenite-induced SeP co-localizes with lysosomal marker LAMP2, and the induced SeP was lowered by SFN treatment gradually from 2 to 6 µM (Fig. 2a and Supplementary Fig. S3). When LAMP2 levels were kept constant, the number of lysosomes did not seem to be affected by SFN treatment. To detect acidic lysosomes, we used LysoTracker, which is a cell-permeable fluorescent acidotropic probe for labeling acidic organelles in live cells. Unlike LAMP2, LysoTracker almost failed to detect acidic-lysosome at untreated SFN, and LysoTracker positive dots were increased by SFN treatment (Fig. 2b). These results suggest that SFN affects not the number of lysosomes, but rather a lysosomal function. *V-ATP A1*, a subunit of vacuole-type ATPase that is essential for the acidification of lysosomes, was also up-regulated at the mRNA level when treated with 6 µM SFN (Fig. 2c), indicating that lysosomal function was activated by SFN. To investigate the involvement of SFN in the reduction of SeP via lysosome, we treated HepG2 cells with inhibitors of lysosomal acidification, chloroquine (CQ) or bafilomycin A1 (BafA1)^28,29^, and examined the effects. The inhibitory action of CQ or BafA1 was confirmed by increased expression of LC3-II and p62, which are specifically degraded by lysosomes (Fig. 2d, e). p62 was increased by SFN and further increased with co-treatment of CQ or BafA1, suggesting that SFN increased p62 via Nrf2 activation and then CQ or BafA1 stopped its degradation in the lysosome. Under these conditions, CQ or BafA1 restored SeP to levels similar to the control (Fig. 2d, e). These results indicate that SFN enhanced protein degradation of SeP in lysosomes. Since CQ or BafA1 did not affect the amount of extracellular SeP secreted to the medium, it is considered that inhibition of lysosomal degradation only affects intracellular SeP levels.

### Nrf2 activation by SFN is not involved in the downregulation of SeP at the protein level

**Fig. 3:**
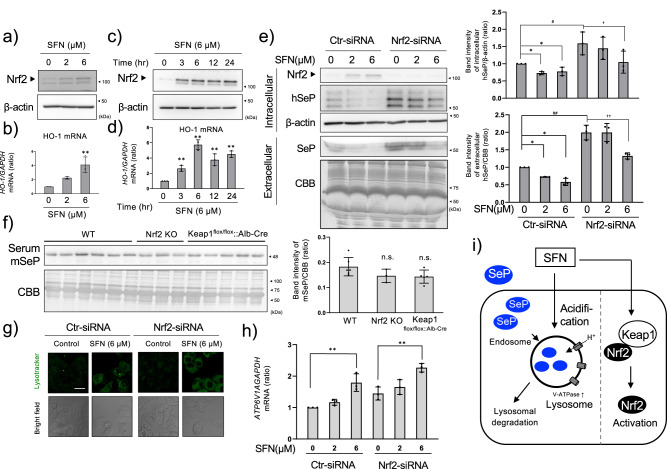
Involvement of Nrf2 activation in the reduction of SeP by SFN. HepG2 cells were cultured in high glucose DMEM containing selenite (100 nM) for 24 h and treated with SFN. SFN concentration-dependency of Nrf2 activation for 24 h (**a**), and *HO-1* mRNA expression level (**b**) or time-dependency of Nrf2 activation with SFN at 6 µM (**c**) and *HO-1* levels (**d**) are shown. Control and *Nrf2* siRNA were transfected into HepG2 cells and cultured for 24 h. The cells were treated with SFN at the indicated concentrations for 24 h. Cell lysate and culture supernatant were collected, and western blotting was performed (**e**). Representative blotting (left panel) and quantifications of the band intensity (right panel, *n* = 3) are shown. WT, Nrf2 KO, Keap1^flox/flox::^Alb-Cre mouse serum was collected and SeP was detected by western blotting (**f**, quantifications of the band intensity are shown in the right panel (WT *n* = 6, Nrf2 KO *n* = 3, and Keap1^flox/flox::^Alb-Cre *n* = 5). Control and *Nrf2* siRNA were introduced into HepG2, cultured for 24 h, and treated with SFN at the indicated concentrations for 24 h. After that, the cells were stained with Lysotracker and observed with a confocal microscope (**g**). The scale bar indicates 20 µm. Under the same conditions, *ATP6V1A* mRNA was measured by RT-qPCR (**h**). The scheme of the underlying mechanism suppressing SeP expression by SFN is shown (**i**). The data is expressed as mean ± S.D. (*n* = 3) and indicated as a relative value with the control as 1. *P < 0.05 and **P < 0.01 vs. control. One-way ANOVA, post hoc test Dunnett method were used for statistical analysis. All blots were performed on independent membranes and were done with the same sample volume applied. CBB staining was performed on the same membrane.

As an Nrf2 activator, SFN is expected to activate lysosomes via Nrf2^30^. Indeed, SFN activated Nrf2 (Fig. 3a, c) and induced gene expression of its downstream gene, heme oxygenase-1 (*HO-1*) (Fig. 3b, d), in time- and dose-dependent manners (Fig. 3a–d). However, the decrease of SeP by SFN was not canceled by *Nrf2* siRNA (Fig. 3e), suggesting that SFN-induced Nrf2 activation is not involved in the decrease of SeP at the protein level. Unexpectedly, *Nrf2* knockdown enhanced basal *SELENOP* mRNA and protein levels under these conditions (Supplementary Fig. S4). Transfection of multiple siRNAs against *Nrf2* also enhanced intracellular SeP at the protein level (Supplementary Fig. S5), thus these data suggest that SeP at the protein expression level is negatively regulated by basal Nrf2 activation in HepG2 cells. In addition, SeP expression at the protein level was reduced with *Nrf2* siRNA treatment, though *SELENOP* mRNA expression was retained (Fig. 3e and Supplementary Fig. S4). These data support the hypothesis that SFN lowers SeP at the protein level, but not at the transcription level. Serum SeP levels were not affected by knockout of Nrf2 or liver-specific knockout of Keap1 (Keap1^flox/flox^::Alb-Cre), a suppressor of Nrf2 (Fig. 3f), thus basal Nrf2 activation seems to be irrelevant to extracellular SeP expression in vivo. Lysosomes detected by Lysotracker showed that lysosome function activated by SFN (Fig. 3g), and induction of V-ATPase A1 by SFN was not abolished by *Nrf2* siRNA (Fig. 3h). These data suggest that SFN enhances acidification of lysosomes, resulting in a decrease of SeP in an Nrf2-independent manner (Fig. 3i).

### SFN suppresses serum SeP at the protein level but not at the mRNA level in mouse livers

**Fig. 4:**
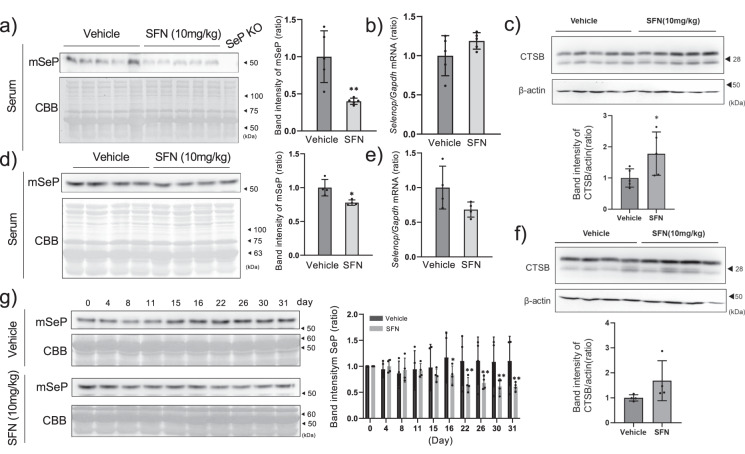
Effect of SFN on serum SeP levels of normal and diabetic mice. C57BL6/J mice were administered 10 mg/kg SFN every 12 h for 48 h. Serum was taken from 5 mice administered either vehicle or SFN, and the amount of SeP was measured by western blotting and quantified (**a**). The serum of a SeP KO mouse was loaded as the negative control (SeP KO). Hepatic *Selenop* mRNA was measured by RT-qPCR (**b**), and mature-cathepsin B (CTSB) protein levels were measured by western blotting (**c**). The SeP band intensity was corrected and quantified by CBB-stained total proteins. The data is expressed as mean ± S.D. (*n* = 5), and shown as a relative value with the control as 1. The student’s *t*-test was used for statistical analysis. KKAy mice were administered 10 mg/kg SFN or vehicle every day for 1 month. Serum was taken from 5 mice per group, and the amount of SeP protein was measured by western blotting (**d**) and its quantification is shown (right panel). The student’s *t*-test was used for statistical analysis. Hepatic *Selenop* mRNA (**e**) and CTSB protein levels were measured (**f**). The change in serum SeP level due to treatment with SFN was measured by western blotting (**g**). The quantified data is expressed as mean ± S.D. (*n* = 4), and shown as a relative value with the control as 1. *P < 0.05 and **P < 0.01 vs. control. All blots were performed on independent membranes and were done with the same sample volume applied. CBB staining was performed on the same membrane.

To evaluate the effects of SFN in vivo, we administrated SFN (10 mg/kg every 12 h for 48 h) intraperitoneally to C57BL6/J mice. SFN significantly reduced mouse SeP (mSeP) levels in the serum without affecting Selenop mRNA levels in the liver (Fig. 4a, b). Under these conditions, the protein level of mature-cathepsin B in the liver was significantly increased by SFN (Fig. 4c), however, mRNA expression of Atp6v1a was not changed (Supplementary Fig. S6). As the acidic conditions of lysosomal maturation trigger cathepsin activation, these results indicate that lysosomal activation induced by SFN contributes to the decrease of SeP at the protein level, similar to the process in HepG2 cells.

Next, we examined whether SFN suppresses SeP in KKAy mice, a representative model of diabetes. The basal levels of serum SeP in KKAy mice tended to increase when compared to C57BL/6 J mice when treated with high glucose (Supplementary Fig. S2) as we reported previously^10^. The molecular size of SeP varied due to glycosylation between wildtype mice in the background of C57BL6/J,ICR, and KKAy, but were made uniform by de-glycosylation(Supplementary Fig. S2). Injection of 10 mg/kg SFN every day did not alter serum SeP levels within 48 h, but gradually decreased after 17 days (Fig. 4d, g). At the endpoint, the hepatic mRNA of Selenop did not change much compared to the vehicle group (Fig. 4e). The protein level of mature-CTSB was partially increased by SFN, however, it was not significant (Fig. 4f). Under the above conditions, hepatic GPx1 levels were increased by SFN in C57BL6/J mice, but not in KKAy mice (Supplementary Fig. S7). These results indicate that responsibility against SFN was found to be lower in diabetic mice, hence blood glucose level and glucose tolerance test (GTT) would not be changed by SFN in the condition (Supplementary Fig. S8).

The increase of antioxidant systems such as glutathione (GSH) may hinder the effect of SFN^31^, since SFN itself is metabolized via GSH conjugation. We then considered the possibility that the high GSH levels in KKAy mice might have prevented the effects of SFN. The results indicate that the hepatic GSH content of normal C57BL6/J wildtype mice was significantly lower than that of KKAy mice (Supplementary Fig. S9a). In addition, forced up-regulation of GSH by N-acetylcysteine (NAC) canceled the decrease of SeP (and Nrf2 activation) by SFN in HepG2 cells (Supplementary Fig. S9b). These results suggest that although SFN can decrease serum SeP levels in mice, it has less effect on mice that contain high GSH levels in the liver.

## Discussion

The present study reports a mechanism by which, (1) SFN reduces selenite-induced SeP transcriptional activation but its effect on SeP protein expression is partial, and (2) SFN mainly promotes SeP breakdown in lysosomes. These results also suggest that (3) activation of the transcription factor Nrf2, a well-known target of SFN, is not involved in the reduction of SeP at the protein level. We found that the suppressive effects of SFN on SeP levels are limited in diabetes model mice, suggesting that high GSH levels in the liver may be one of the reasons limiting SeP reduction by SFN. These findings indicate the health-promoting effects of SFN, but also suggest limitations when applied to therapeutics, and provide new insights from both biological and pharmacological perspectives.

As the isothiocyanate group of SFN covalently binds to nucleophilic thiol and amino groups, it would also bind to the selenol group of highly nucleophilic selenocysteine residues^17^. When SeP bound to SFN via the selenol group is degraded into amino acids, including selenocysteine in lysosomes, the removal of inorganic selenium by selenocysteine lyase may be inhibited. To elucidate this point, it will be necessary to detect the covalent bond between the selenocysteine residue of SeP and SFN. In HepG2, SeP mRNA and protein levels were both decreased by SFN, while in the mouse liver, SeP mRNA was not changed and only the protein levels decreased (Figs. 1c and 4b). Although the details of mRNA reduction by SFN in HepG2 cells are unknown, the decrease in SeP at the protein level is probably due to accelerated proteolysis rather than mRNA reduction both in vivo and in HepG2 cells. In addition, the marker protein used for lysosomal activation by SFN was different in vivo and in HepG2 cells. Lysosomal acidification is determined not only by activation of lysosomal biogenesis, but also by activations/modifications of V-ATPase and counter ion channels. Hence, a comprehensive approach would be helpful to study lysosomal activation by SFN in detail.

Broccoli sprouts contain around 1.9-3.7 mg/g SFN^17^. However, intraperitoneal injection of SFN at doses of 10 mg/kg was required to reduce serum SeP expression in mice, thus these results cannot simply be extrapolated to humans who would ingest it orally^32^. A human weighing at least 60 kg would need to ingest more than 300–600 g of broccoli sprouts per day, making it unrealistic to attempt to reproduce the same effect by dietary means. We also studied oral administration of SFN in mice (30 mg/kg, 4 doses every 12 h) and found it had no effect on serum SeP levels; though the intestinal absorption rate of SFN has not been reported, oral administration of SFN from dietary sources would probably not be enough to cause a significant decrease in SeP. Therefore, to apply the findings of this study to the prevention of diabetes, it will be necessary to find better-absorbed and more effective derivatives of SFN by structural expansion.

SFN has been reported to reduce diabetes-associated dysfunctions via activation of Nrf2, thus there is no doubt that SFN prevents diabetic symptoms via Nrf2 activation, and our findings did not compete with previous reports^32,33^. The Nrf2-independent mechanism of action by SFN has been focused on in recent years. A recent study suggests that SFN inhibits SREBP-1 and this action is independent of the Keap1-Nrf2 pathway. SFN is known to activate transcription factor EB (TFEB), a master regulator of lysosomes, via an alternative pathway to Nrf2, through increased mitochondrial reactive oxygen species production^27^. Many lysosomal constituent proteins have a TFEB-binding region (CLEAR) on their promoters, and this transcription factor is responsible for repairing injured lysosomes and promoting lysosomal biogenesis^34^. Therefore, SFN may contribute to the increase in acidified lysosomes via activation of this transcription factor and promote the degradation of SeP. To prove these theories, it will be necessary to examine the effect of SFN on the activation of TFEB and the number of lysosomes by immunostaining for the marker proteins. Although Nrf2 was not involved in the reduction of SeP by SFN, it is suggested that Nrf2 at a steady state may repress SeP transcription in HepG2 cells. Although the detailed mechanism has yet to be elucidated, it has been reported that Nrf2 suppresses the expression of interleukin 6 and 1β by binding to the promoter regions of inflammatory cytokines in an antioxidant-responsive element (ARE) sequence-independent manner^35^, and perhaps such an action contributes to the suppression of SeP expression. In addition, in the activation of Nrf2 by SFN, Nrf2 forms a heterodimer with small Maf proteins in the nucleus and binds to the ARE sequence, thus its action may have differed from that of Nrf2 at steady state^36^. Further studies are needed to assess these mechanisms.

Recently, it has been reported that increased expression of SeP contributes not only to diabetes mellitus but also to the worsening of pulmonary hypertension via the proliferation of vascular smooth muscle^37^. Few drugs are effective in the treatment of pulmonary hypertension, and SeP is likely to be a therapeutic target for this disease^37^. Therefore, the development of drugs based on natural products that reduce the expression of SeP, such as SFN, offers hope to address unmet medical needs, not only in diabetes but also in pulmonary hypertension. The findings of this study provide an important basis for the creation of SeP inhibitors, which will be useful for the future prevention and treatment of SeP-associated diseases.

## Methods

### Cell culture

The cell line used in this study is HepG2 (derived from human liver carcinoma, JCRB cell bank). HepG2 cells were cultured in high glucose DMEM with 10% fetal bovine serum (FBS), 100 U/mL and 100 µg/mL penicillin–streptomycin in a humidified incubator under the conditions of 37 °C, 5% CO_2,_ and 95% ambient air. Selenium levels in FBS used in this study were determined by ICP-MS (Agilent, CA, USA), and 8 µg/L was used. For cell maintenance, HepG2 cells were cultured in a 10 cm dish passaged at a cell density of 10% or 20% and cultured to an 80% subconfluent state. In the present study, HepG2 was seeded 24 h before the experiments.

### LDH assay

HepG2 cells were seeded in 96 well plates and cultured for 24 h, then treated with SFN by exchanging the medium with fresh medium containing SFN (high glucose DMEM, 10% FBS, 1% penicillin–streptomycin solution) at the indicated concentration. After 24 h, the cytotoxicity was measured by the LDH Cytotoxicity Detection Kit according to the manufacturer’s protocol (Takara, Shiga, Japan).

### SDS-PAGE and western blotting

To obtain whole-cell lysates, cells were suspended in lysis buffer [16 mM Tris-HCl (pH7.5), 150 mM NaCl, 1% NP-40, 0.1% SDS, 1% sodium deoxycholic acid with a cocktail of protease inhibitors (Nacalai Tesque, Kyoto, Japan)] at 4 °C for 30 min. Nuclei and insoluble cellular debris were removed by centrifugation at 15,000 × *g* for 5 min. The protein concentration was determined using a DC protein assay kit (Bio-Rad, CA, USA) with bovine serum albumin as the standard. Total protein in the medium was detected by Coomassie brilliant blue G-250 (CBB). The protein samples were separated by sodium dodecyl sulfate-polyacrylamide gel electrophoresis (SDS-PAGE) and subjected to western blotting with the indicated antibodies. The antibodies used in this study are listed in Supplementary Table 1. Chemiluminescence was detected using Chemi-Lumi One (Nacalai) and a chemiluminescence detector (Atto, Tokyo, Japan). Protein size was noted by comparing it to the molecular weight marker colored on the membrane at the time of WB detection. For some data, size markers and merge photos are shown in the uncropped data in the supplemental data.

### Reverse transcription-quantitative PCR (RT-qPCR)

Total RNA was extracted with ISOGEN II (Nippon Gene, Tokyo, Japan). The obtained RNA was quantified using nanodrop ND-1000 (Thermo) and was subjected to a reverse transcription reaction using Prime Script (Takara) according to the attached instructions to obtain cDNA. Primers against the gene of interest were diluted to 10 µM using nuclease-free water (Qiagen, Hiledn, Germany). The sampled cDNA stock solution was diluted 10-fold and used as the template. Template 2 µL, POWER SYBR GREEN 10 µL (Thermo), forward and reverse primers (Supplementary Table 2) were added to each well of the 96-well plate and adjusted to a total of 20 µL. Synthesized primers were obtained from Fasmac (Japan, Kanagawa). The solution was then subjected to RT-qPCR under the following conditions using the Thermal Cycle Dice rear time system (Takara). *GAPDH* was used as the internal standard target gene.

### Immunocytochemistry and lysotracker assay

HepG2 cells (1.4 × 10^4^ cells) were seeded in an 8-well slide chamber and cultured for 24 h. After that, they were treated with 0, 2, and 6 µM SFN for 24 h, washed with PBS, fixed with 4% paraformaldehyde (PFA) for 5 min, washed once with PBS, treated with 0.1% Triton X-100 for 5 min for permeabilization, and washed with PBS again. The cells were then blocked with 1% BSA/PBS for 30 min, washed with PBS, and soaked with anti-LAMP2 antibody (2 µg/mL; Santa Cruz Biotechnology) diluted with 1% BSA/PBS, then incubated overnight. After washing with PBS, the secondary antibody with Alexa Fluor 488 (anti-mouse, Abcam) was diluted 2000-fold with 1% BSA/PBS and reacted for 2 h. The cells were washed with PBS and soaked in anti-hSeP antibody BD1 (2 µg/mL) diluted with 1% BSA/PBS for 2 h. After washing with PBS, the secondary antibody with fluorophore Alexa Fluor 594 (anti-rat, Abcam) was diluted 2000-fold with 1% BSA/PBS, and treated for 1 h. After that, the slide was encapsulated with VECTASHIELD HardSet with 4’,6-diamidino-2-phenylindole (DAPI). Fluorescence was observed with a confocal microscope FV1000 (Olympus, Tokyo, Japan). Filters were set to Ex: 590 nm, Em: 617 nm for detecting SeP, and Ex: 495 nm, Em: 519 nm for LAMP2 and Ex: 360 nm, Em: 460 nm for DAPI.

For the lysotracker experiment, the cells were seeded in the slide chamber and then treated with 0, 2, and 6 µM SFN for 24 h. The culture medium was replaced with fresh medium containing 75 nM Lysotracker Green DND-26 (Thermo) and incubated at 37 °C for 30 min. After replacement with the new medium, fluorescence was observed by confocal microscope FV1000 (Olympus) (Lysotracker, Ex: 504 nm, Em: 511 nm).

### Transfection of siRNAs

A transfection complex (Opti-MEM 500 µL, 10 µM siRNA 3 µL, Lipofectamine® RNAiMAX reagent 3 µL) was prepared and added to the culture medium. The cells were further cultured at 37 °C, 5% CO_2_, and 95% ambient air for 24 h. The sequences of siRNAs used in the study are listed in the supplemental information. Four types of siRNAs, which target different sites of *Nrf2* mRNA, were used and we checked that each siRNA gave the same phenotypes (Supplementary Fig. S5). We used si*Nrf2* #1 as representative siRNA because suppressed *Nrf2* mRNA significantly and stably. The control siRNA used was SIC-001, a Universal Negative siRNA manufactured by Sigma.

### Mouse

The animal study was carried out in accordance with the rules and guidelines for the proper implementation of animal experiments at Tohoku University and the experimental plan was approved by the Support Center for Laboratory Animal and Gene Researches, Tohoku University (Approval No. 2019-018-05). Nrf2 knockout and liver-specific Keap1 knockout mice (Keap1^flox/flox^::Alb-Cre) were reported previously^38–40^. SeP knockout mice were reported previously as well^41^.

SFN suspended in 0.9% NaCl was intraperitoneally administered to male 6-week-old C57BL/6J mice (Claire Japan) that had been acclimatized for 1 week. Equal doses of 0.9% NaCl were administered to the vehicle group. Sampling was performed after a total of 4 doses for 48 h every 12 h. After inhalation anesthesia with isoflurane, blood was collected from the left ventricle with a syringe, centrifuged at 900 × *g* for 10 min, and the supernatant was collected to obtain a serum sample. Next, the abdominal vena cava was amputated, and reflux was performed with PBS from the left ventricle. The liver was then removed and frozen in liquid nitrogen. Approximately 20–30 mg of liver were collected, homogenized in 500 μL of tissue extraction buffer using a polytron homogenizer, centrifuged at 8000 × *g* at 4 °C for 5 min, and the supernatant was collected as a protein extract. Serum and organ protein concentrations were quantified using the DC protein assay kit (Bio-Rad). mRNA was extracted from the liver by Isogen II. The obtained RNA was measured for concentration and purity by NanoDrop (Thermo).

Male 16-week-old KKAy mice (CLEA Japan, Kanagawa, Japan) were intraperitoneally administered 10 mg/kg SFN suspended in 0.9% NaCl every day for 1 month. Blood was collected from the tail every day after treatment with SFN and blood glucose level was measured by LAB Gluco (Foracare, Tokyo, Japan).

### Statistics and reproducibility

For statistical processing, Student’s *t*-test was used for comparison between the two groups. A multiple comparison test (one-way ANOVA, post hoc test Dunnett method) was used to detect significant differences between three or more groups. The difference was significant when the risk level was 5% or less. The data are expressed as mean ± standard deviation (S.D.). At least three replicates (biological replicates) were obtained with independent experiments. Detailed sample sizes are described in the figure legends. Although statistical information on fluorescent staining is not included, representative results of the three independent experiments are indicated.

### Reporting summary

Further information on research design is available in the Nature Portfolio Reporting Summary linked to this article.

### Supplementary information

supplemental information

Supplementary Data 1

Description of Additional Supplementary Data

Reporting summary

## Data Availability

All of the uncropped data are shown in supplementary information Fig. S10–18. All data used for generating graphs in the manuscript are shown in Supplementary Data 1. Source data are obtained as follows. western blotting images were obtained by Image Saver 6 (ver 2.7.2, ATTO), and RT-qPCR data were obtained by Thermal Cycler Dice Real Time System Single Software (ver 5.11, Takara). Microscopy images were obtained by FV-10 ASW (Ver 1.6, Olympus). The data obtained by plate reader were from Soft Max Pro 7 (ver 7.1, Molecular device). All other data are available from the corresponding author (or other sources, as applicable) on reasonable request.
