# Supplementary material for: Sulforaphane decreases serum selenoprotein P levels through enhancement of lysosomal degradation independent of Nrf2
Source: Commun Biol. 2023 Oct 19;6:1060. doi: 10.1038/s42003-023-05449-y (PMC10587141; doi:10.1038/s42003-023-05449-y)
Supplement: Supplementary file 1 — supplemental information [file 42003_2023_5449_MOESM1_ESM.pdf]

Supplemental materials and figures

Sulforaphane decreases serum selenoprotein P levels through the enhancement of lysosomal degradation independent of Nrf2

1. Supplemental Materials and Methods

1.1. Transfection of siRNAs

A transfection complex (Opti-MEM 500 µL, 10 µM siRNA 3 µL, Lipofectamine® RNAiMAX reagent 3 µL) was prepared and added to the culture medium. Then the cells were further cultured at 37 °C., 5% CO2, and 95% ambient air for 24 hours. The sequence of siRNA used is as follows (synthesized by Sigma).

si Nrf2 # 1 F: CAAACAGAAUGGUCCUAAA
R: UUUAGGACCAUUCUGUUUG
2 F: CUCACAAGAGAUGAACUUA
R: UAAGUUCAUCUCUUGUGAG
3 F: GCUCAUACUUUAUAAGUAA
R: UUACUUAUAAAGUAUGAGC
4 F: CUGUUGAUUUAGACGGUAU
R: AUACCGUCUAAAUCAACAG

The control siRNA used was SIC-001, Universal Negative siRNA manufactured by Sigma.

1.2 .Deglycosylation assay

Plasma of C57BL6/J, ICR and KKAy mice was treated with deglycosylation according to the protocol of Protein Deglycosylation Mix II (Biolabs, P6044S) and Western blotting was performed.

1.3. Glucose tolerance test

After treatment with SFN for 1 month, KKAy mice were fasted for 14 h, and 0.3 g/kg glucose was intraperitoneally administered. Blood was collected from the tail and glucose levels were measured 0-120 min after glucose treatment using Lab Gluco (RIJ, Japan, Tokyo).

Supplemental Table 1 Antibodies used for Western blotting

**Primary antibody**		
anti-β-actin	A5441, clone AC-15	Sigma-Aldrich MO
anti-GPx1	ab108427	abcam
anti-GPx4	ab125066	abcam
anti-HO-1	sc-390991	Santa Cruz Biotechnology
anti-LAMP2	sc-18822	Santa Cruz Biotechnology
anti-LC-3A/B	12741S	Cell Signaling Technology
anti-Nrf2	sc-365949	Santa Cruz Biotechnology
anti-p62	511S	Cell Signaling Technology
anti-hSeP		Mita et al., 2017
anti-mSeP		Mita et al., 2017
**Second antibody**		
anti-mouse	20051789	Dako-Agilent
anti-rabbit	20073563	Dako-Agilent
anti-rat	00063346	Dako-Agilent

All primary antibody were used 1/1000 dilution, and 2nd antibody were used 1/10000 dilution.

Supplemental Table 2 Primers for RT-qPCR

Human		
ATP6V1A F	F	AGGCTGAAATCGAGAACCCC
	R	GCTCGGAACCCTTCACAGAT
GAPDH	F	GCACCGTCAGGCTGAGAAC
	R	TGGTGAAGACGCCAGTGGA
HO-1	F	CCAGCAACAAAGTGCAAGATTC
	R	TCACATGGCATAAAGCCCTACAG
Nrf2	F	TCCAGTCAGAAACCAGTGGAT
	R	GAATGTCTGCGCCAAAAGCTG
SELENOP	F	CCCCCAGCCTGGAGCATAAG
	R	TGCACAGGTATCAGCTGGCTT
Mouse		
Selenop	F	AGCTCTGCTTGTTACAAAGCC
	R	CAGGTCTTCCAATCTGGATGC

Supplementary Figure 1

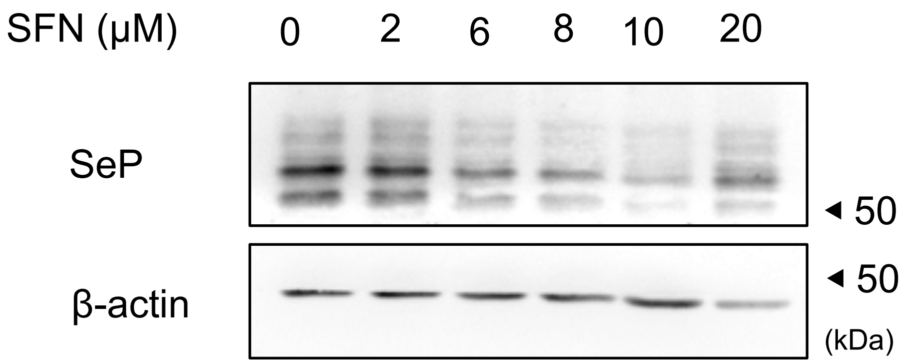

Supplemental Figure 1. Effect of SNF on intracellular SeP at the protein level in a dose-dependent manner.

HepG2 cells cultured with selenite (100 nM) were treated with the indicated concentration of SFN for 24 hr. The cell lysate was then subjected to Western blotting. All blots were performed on independent membranes and were done with the same sample volume apply.

Supplementary Figure 2

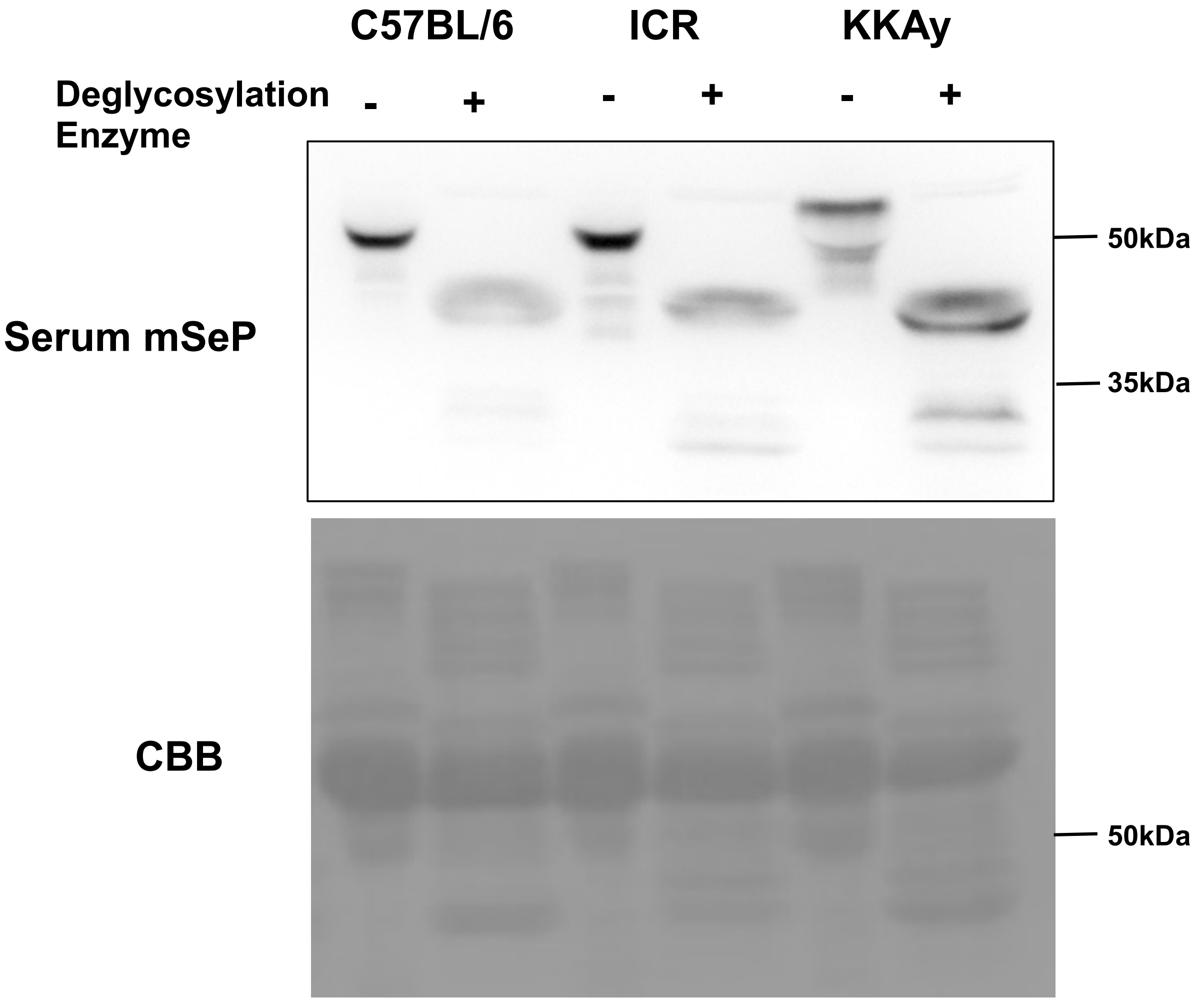

Supplemental Figure 2. Molecular size shift of SeP by glycosylation in different strains of mice. Serum of C57BL6/J, ICR and KKAy mice were treated with deglycosylation according to the Protein Deglycosylation Mix II (Biolabs, P6044S) protocol and WB of SeP was performed.

Supplementary Figure 3

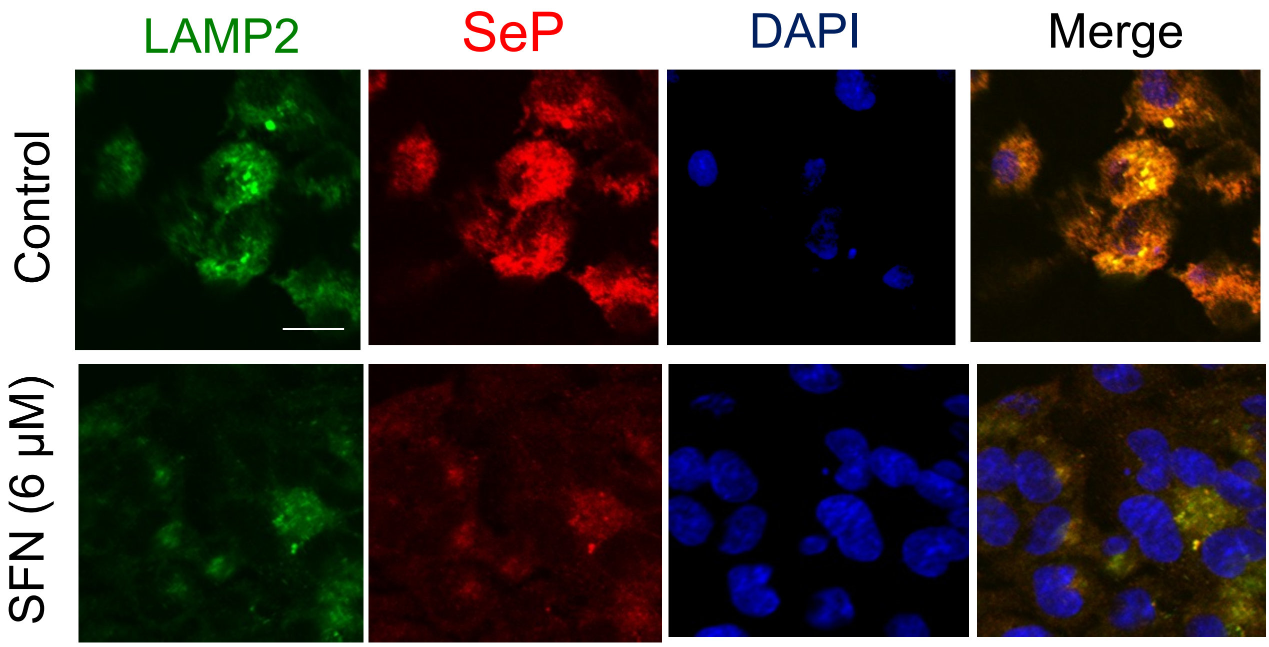

Supplemental Figure 3. Detailed image of Figure 2a.

Magnified images of Figure 2a are shown. The scale bar indicates 20 μm.

Supplementary Figure 4

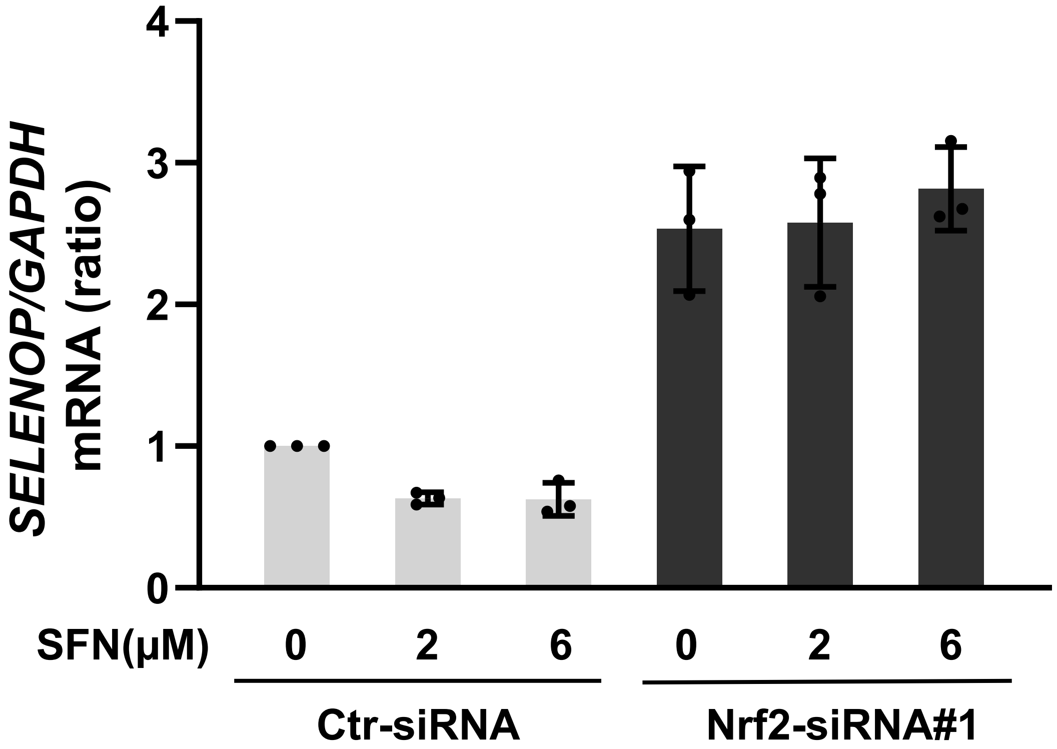

Supplemental Figure 4. Effect of Nrf2 knockdown on SeP mRNA.

Control and Nrf2 siRNA #1 were introduced into HepG2 cells, cultured for 24 hr and treated with SFN at the indicated concentrations for 24 hr. Total RNA was collected and SeP mRNA was measured using RT-qPCR. Data indicates mean ± S.D

Supplementary Figure 5

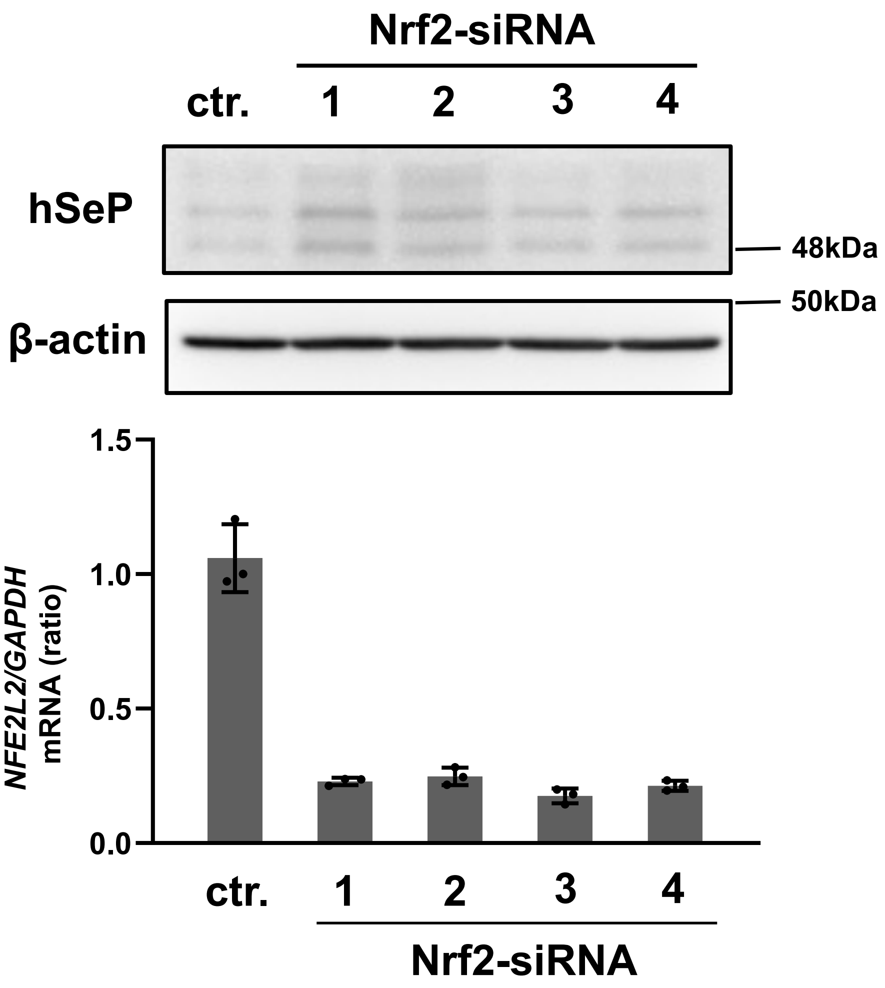

Supplemental Figure 5. Effect of Nrf2 knockdown on SeP protein expression in HepG2 cells. Control and four types of Nrf2 siRNA were transfected into HepG2 cells and cultured for 24 hr. Cell lysates were collected, and WB was performed (upper panel). Knockdown efficiency was examined using RT-qPCR (lower panel). Data indicates mean ± S.D. All blots were performed on independent membranes and were done with the same sample volume apply.

Supplementary Figure 6

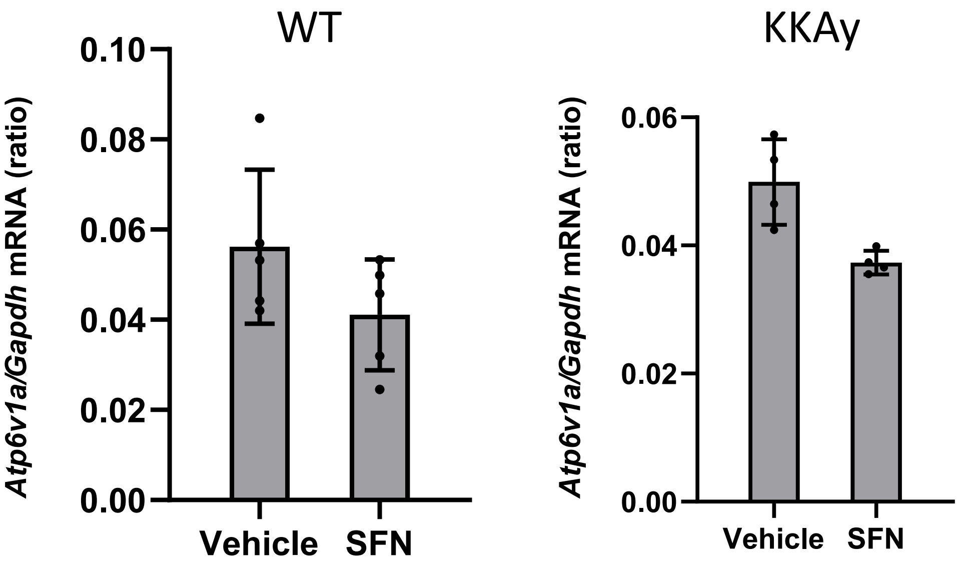

Supplemental Figure 6. Effect of SFN on the mRNA of ATP6V1A in mice liver.

C57BL6/J male mice (n=5) were treated with SFN (10mg/kg) by i.p., every 12 hr for 48 hr. mRNA levels of Atp6v1a in the liver were measured by RT-qPCR (left panel). KKAy mice (n=4) were administered 10 mg/kg SFN every day for 1 month. Hepatic Atp6v1a mRNA levels were measured by RT-qPCR. The data is expressed as Mean ± S.D.

Supplementary Figure 7

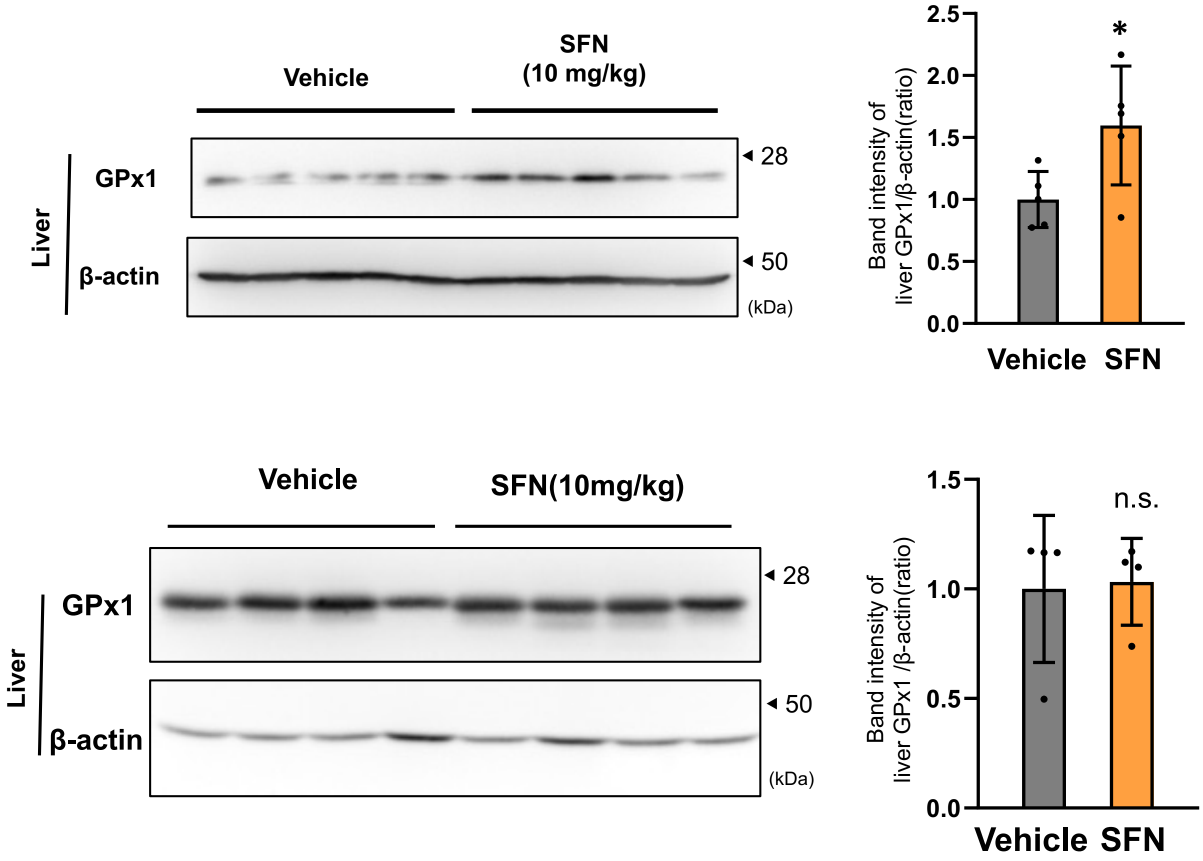

Supplemental Figure 7. Effect of SFN on GPx1 protein and mRNA levels in vivo.

Livers were taken from WT or KKAy mice treated with Vehicle and SFN, and the expression level of GPx1 was measured by WB. The GPx1 band was corrected and quantified with β-actin, expressed as N = 5 or 4, Mean ± S.D., and shown as a relative value with the control as 1. * $P < 0.05$ vs control. All blots were performed on independent membranes and were done with the same sample volume apply.

Supplementary Figure 8

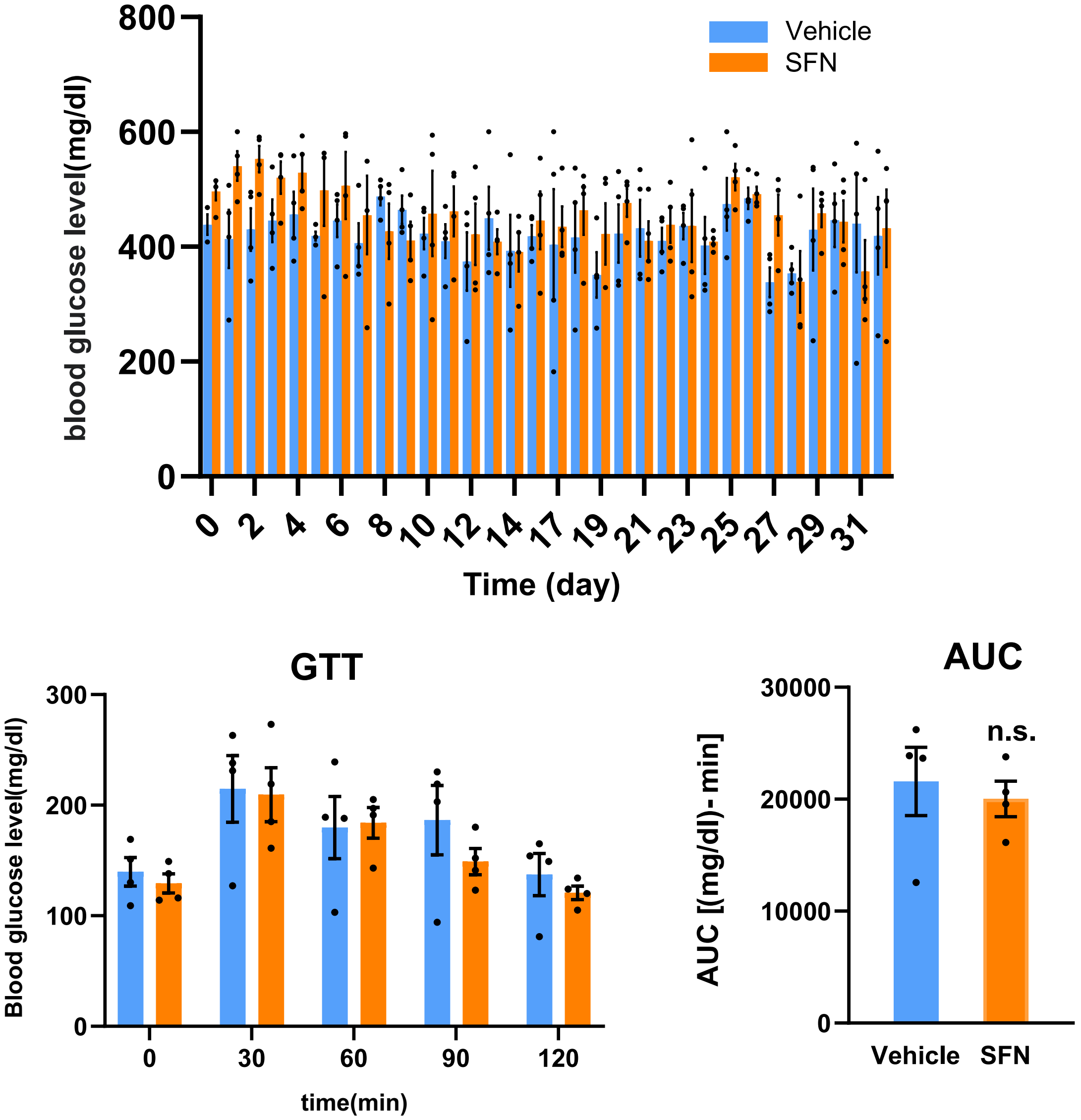

Supplemental Figure 8. Blood glucose levels and glucose sensitivity of diabetic-mice treated with SFN.

The change in blood glucose levels in KKAy mice treated with Vehicle and SFN was measured. A glucose tolerance test was conducted after treatment with SFN.

Supplemental Figure 6. Elevated GSH levels attenuate the effects of SFN

(A)Liver GSH values of normal C57BL6/J mice and diabetic KKAy mice. The quantified data is expressed as N=4, Mean ± S.D., and shown as a relative value with the control as 1. * P <0.05 vs control. (B) Intracellular and extracellular SeP expression of HepG2 cells treated with NAC and SFN for 24hr.

Supplementary Figure 9

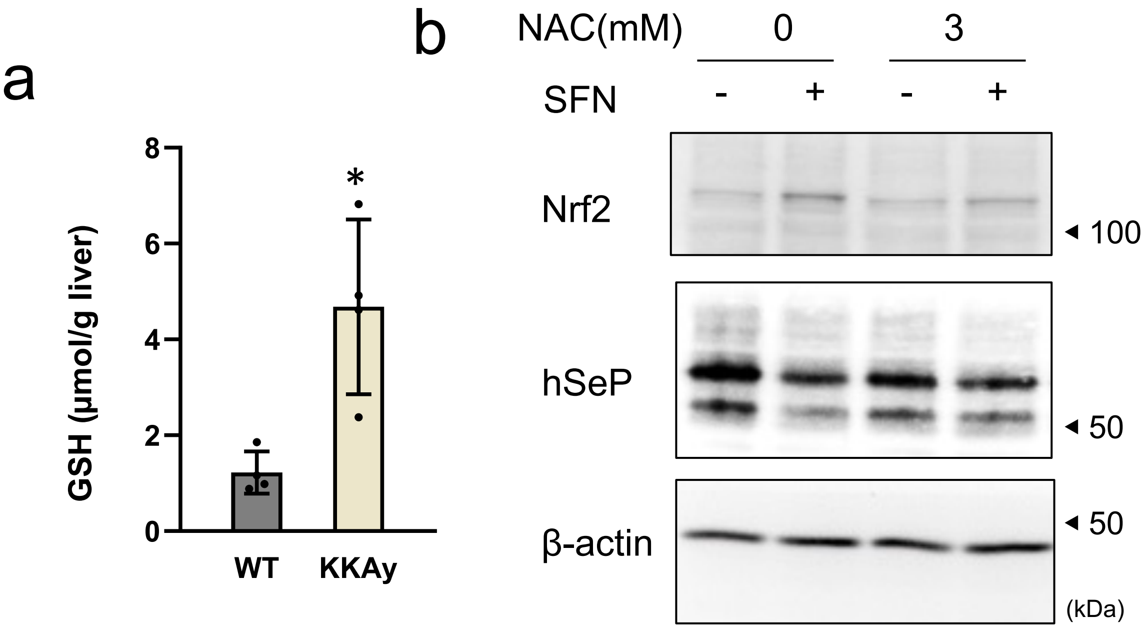

Supplemental Figure 9. Glutathione content in the liver of KKAy mice and the effect of NAC on SeP reduction by SFN in HepG2 cells.

(a) Liver GSH values of normal C57BL6/J mice and diabetic KKAy mice. The quantified data is expressed as N=4, Mean ± S.D., and shown as a relative value with the control as 1. * $P < 0.05$ vs control. (b) Intracellular SeP expression of HepG2 treated with NAC (3 mM) and SFN (6 μM) for 24hr. All blots were performed on independent membranes and were done with the same sample volume apply.

Supplementary Figure 10

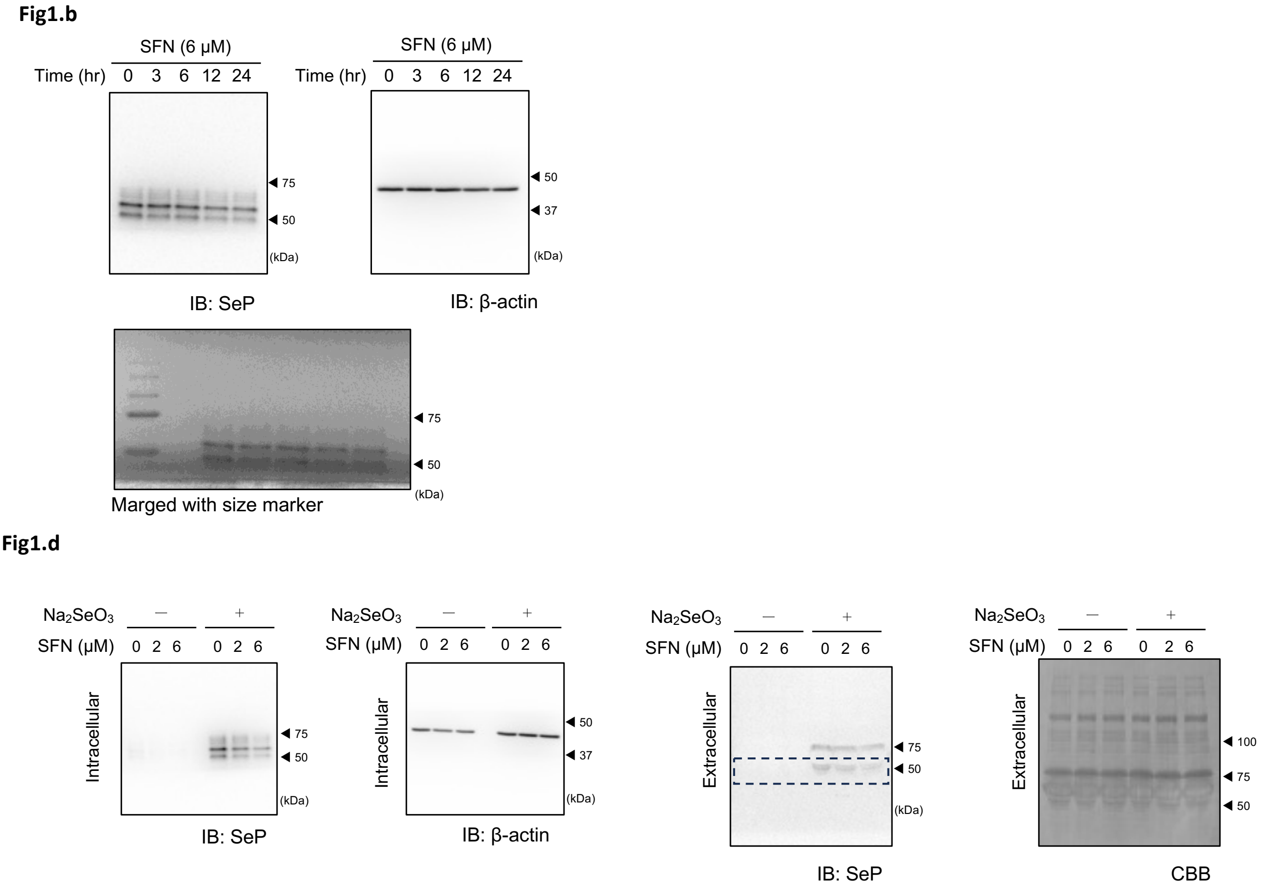

Supplemental Figure 10. Uncropped data of WB figure 1.

Supplementary Figure 11

Fig2.d

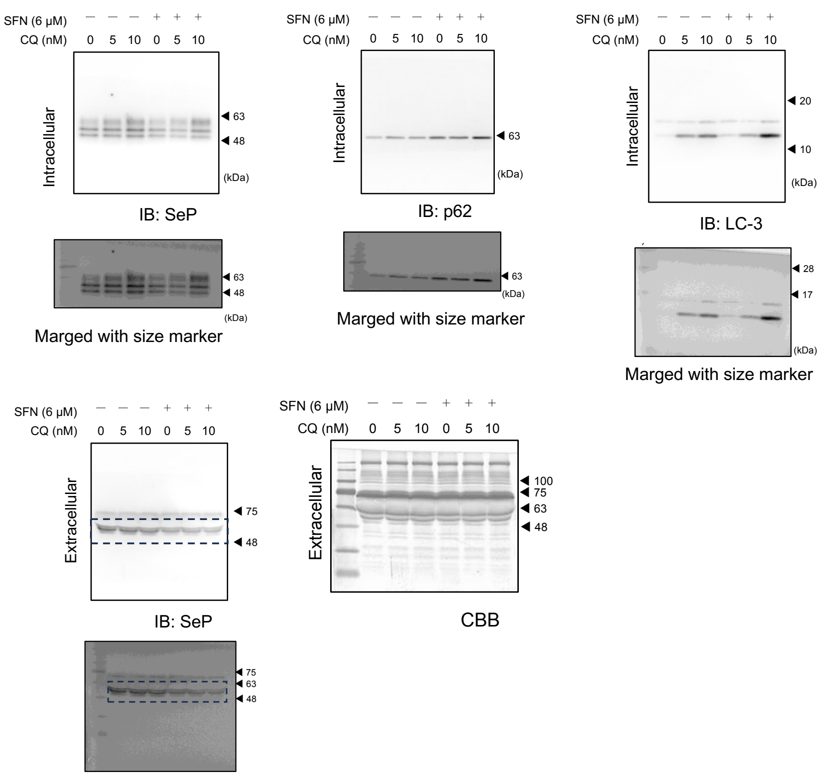

Fig2.e

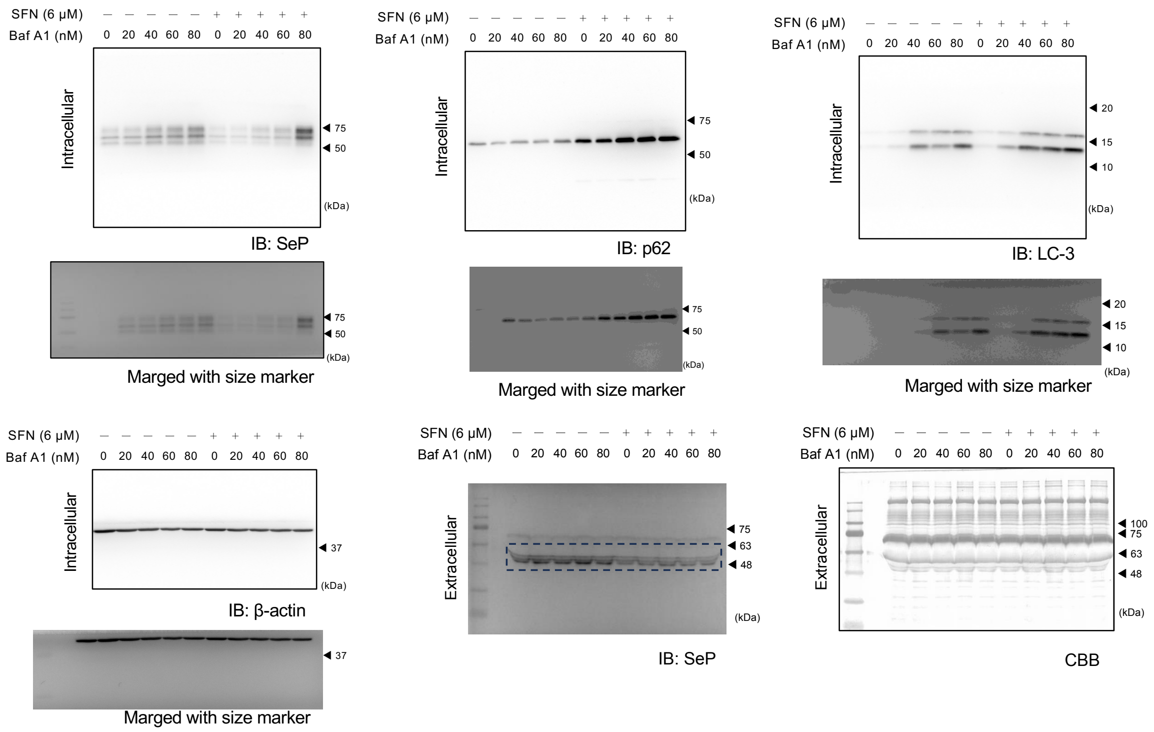

Supplemental Figure 11. Uncropped data of WB figure 2.

Supplementary Figure 12

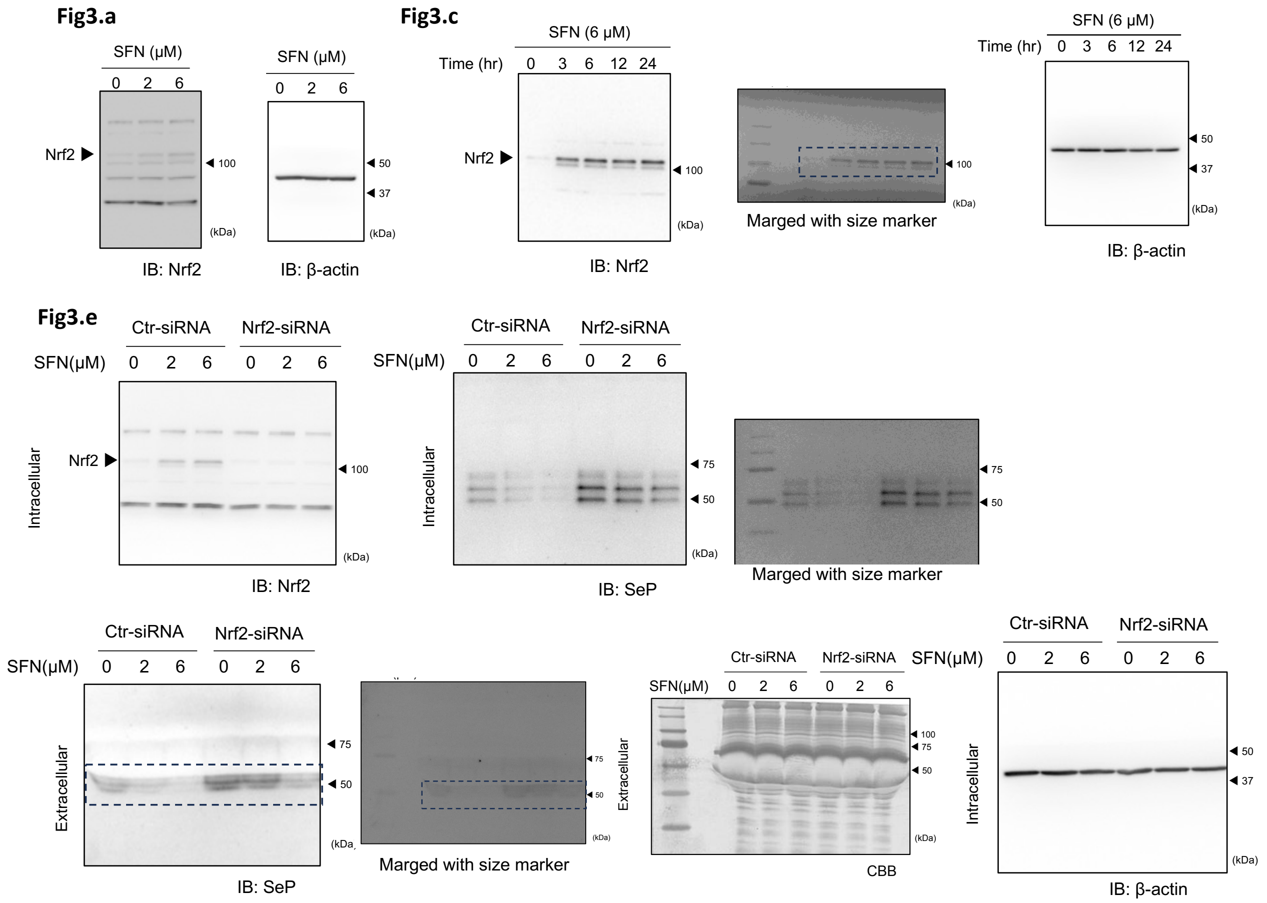

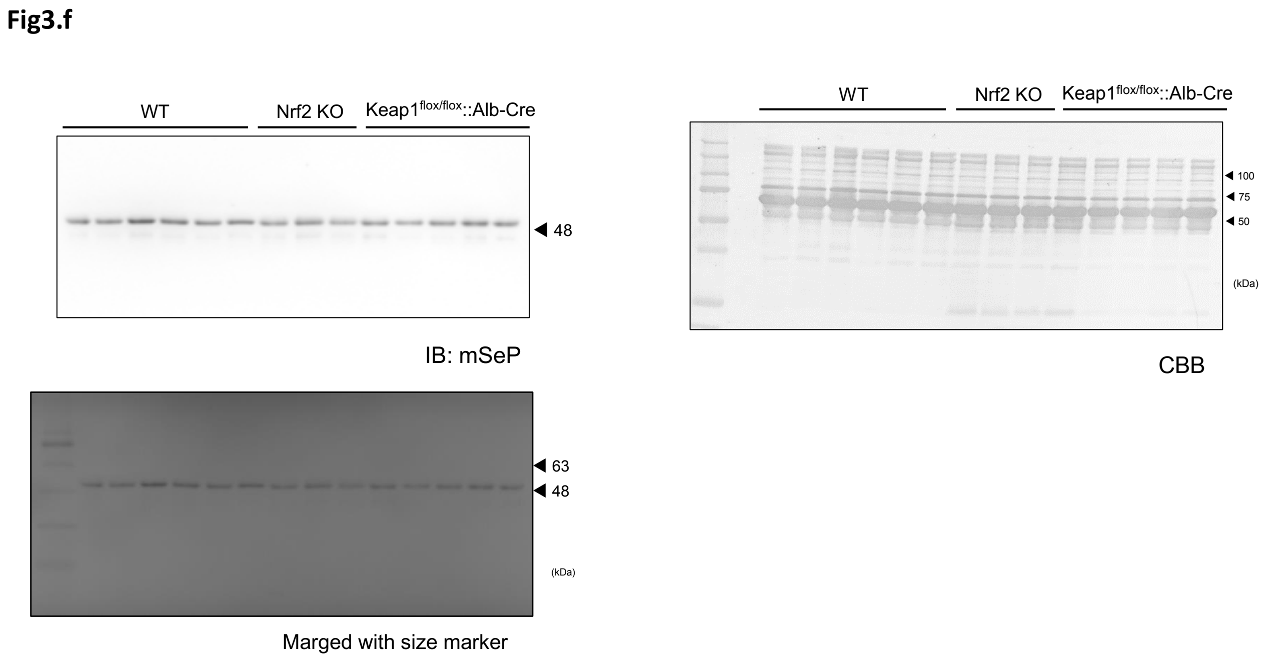

Supplemental Figure 12. Uncropped data of WB figure 3.

Supplementary Figure 13

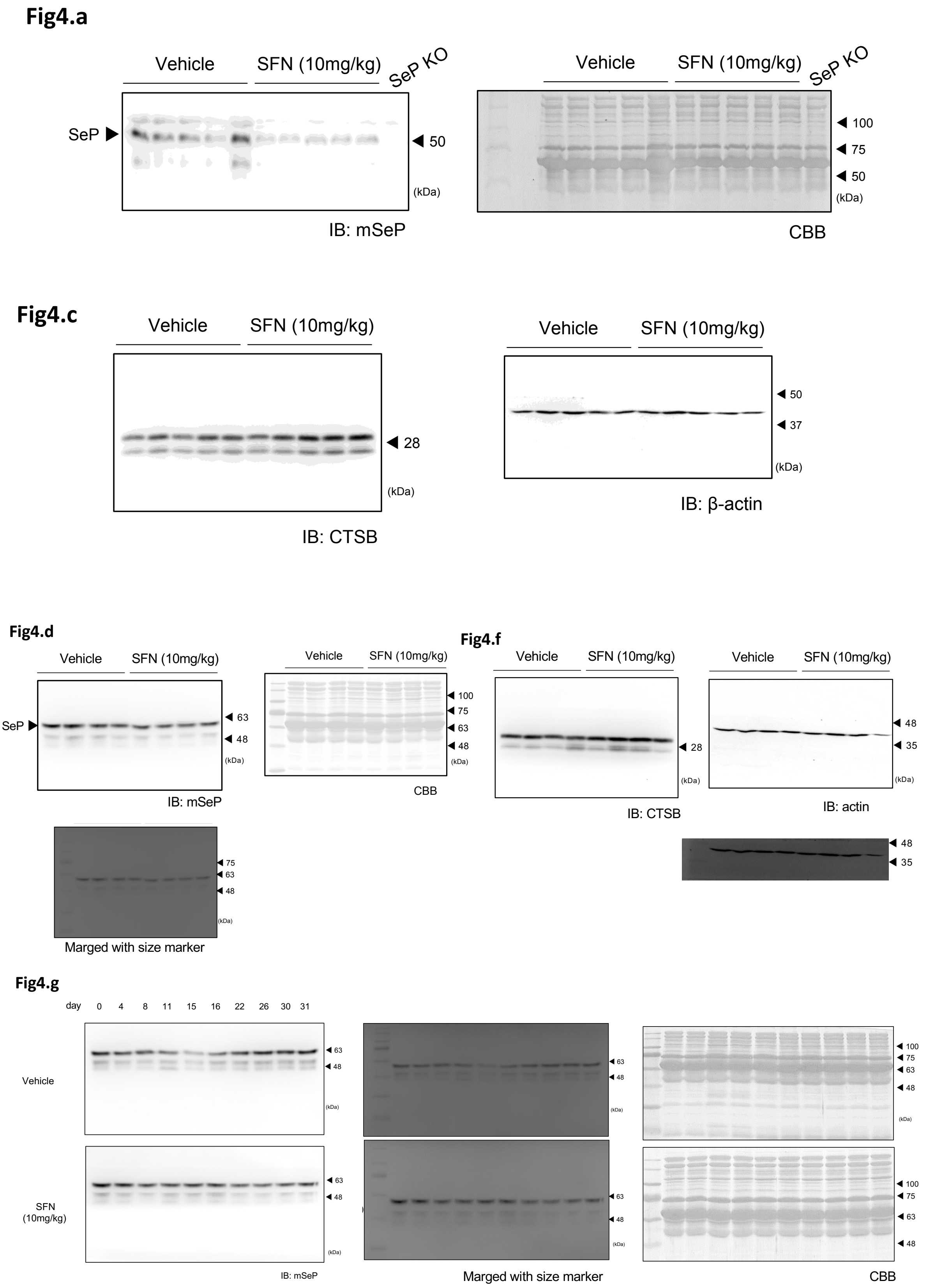

Supplemental Figure 13. Uncropped data of WB figure 4.

Supplemental Figure 1

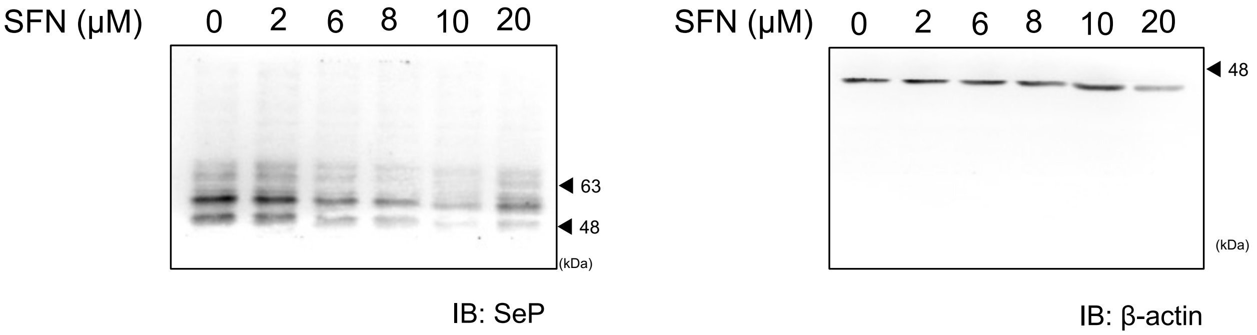

Supplemental Figure 14. Uncropped data of WB S1.

Supplemental Figure 2

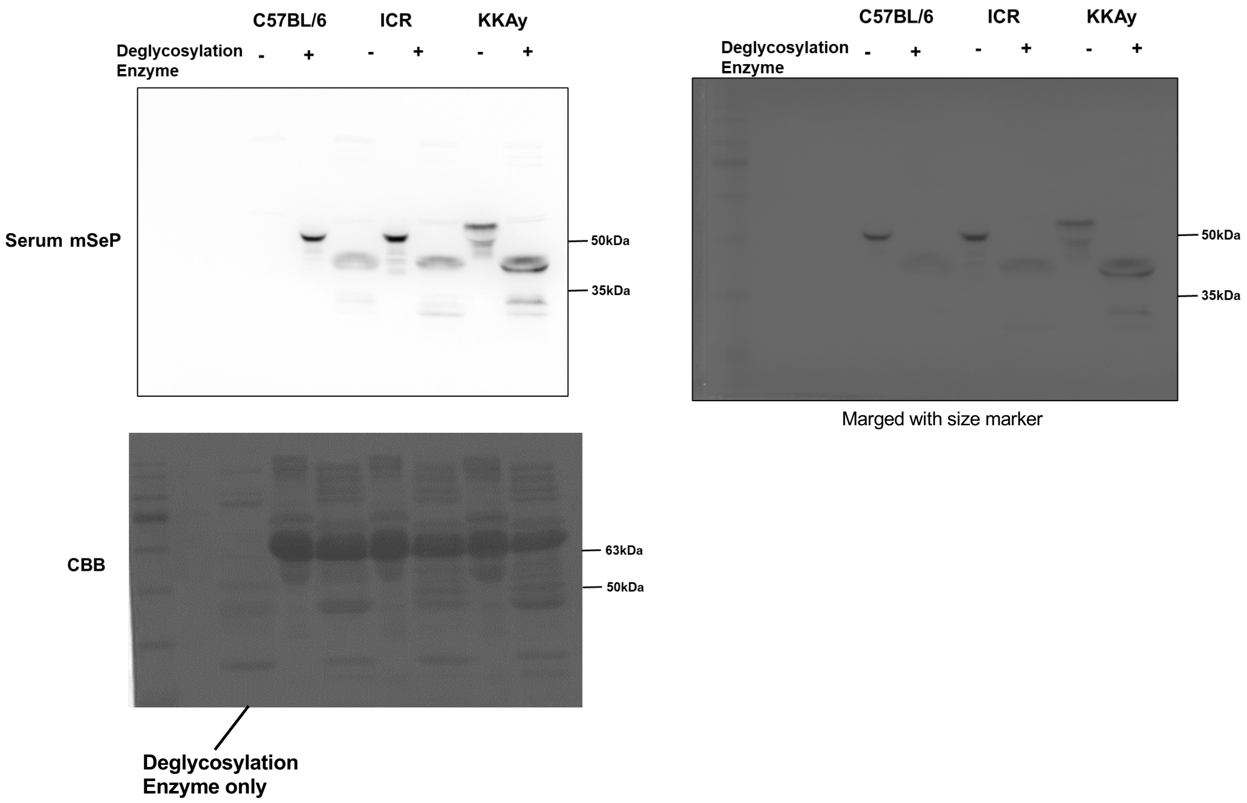

Supplemental Figure 15. Uncropped data of WB S2.

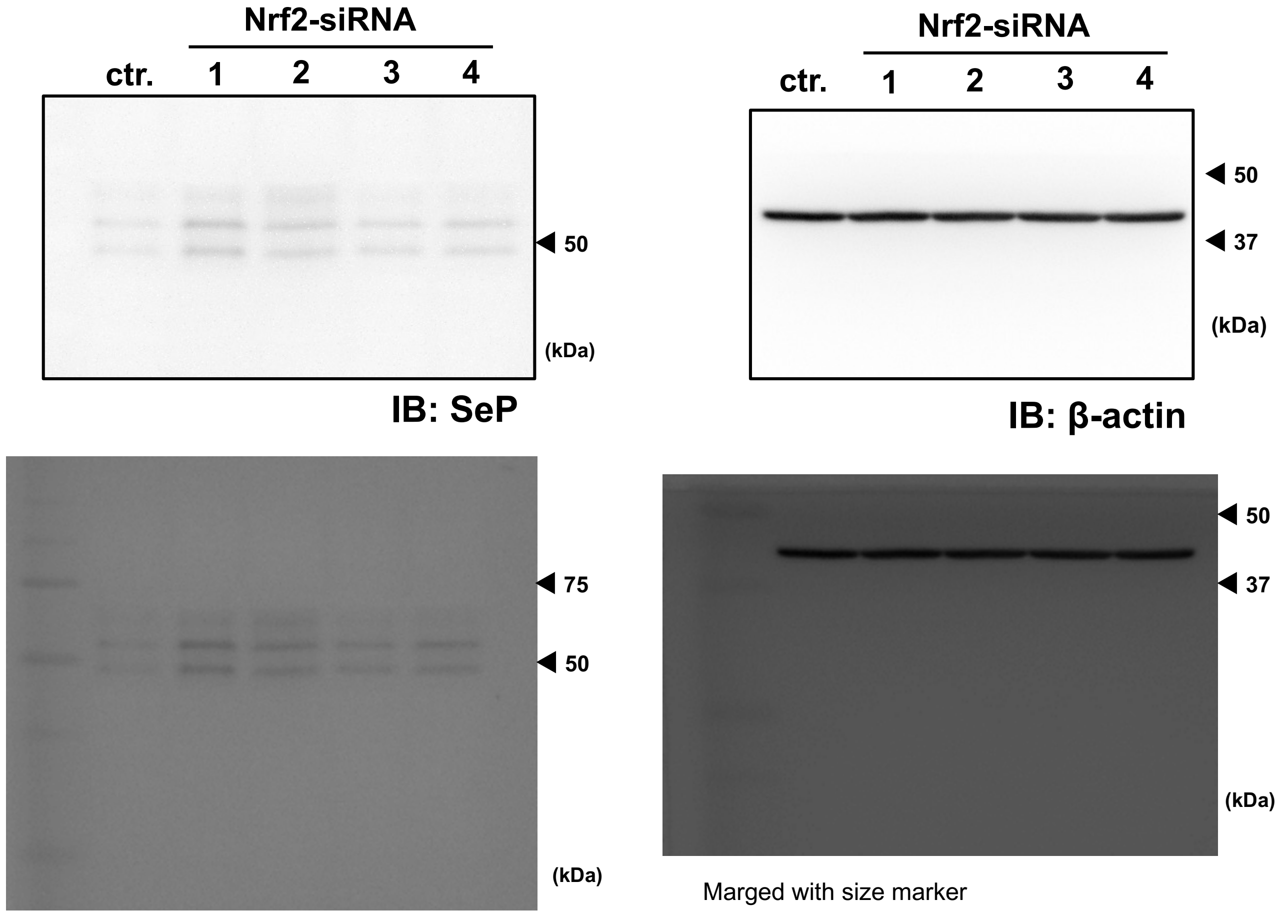

Supplemental Figure 16. Uncropped data of WB S5.

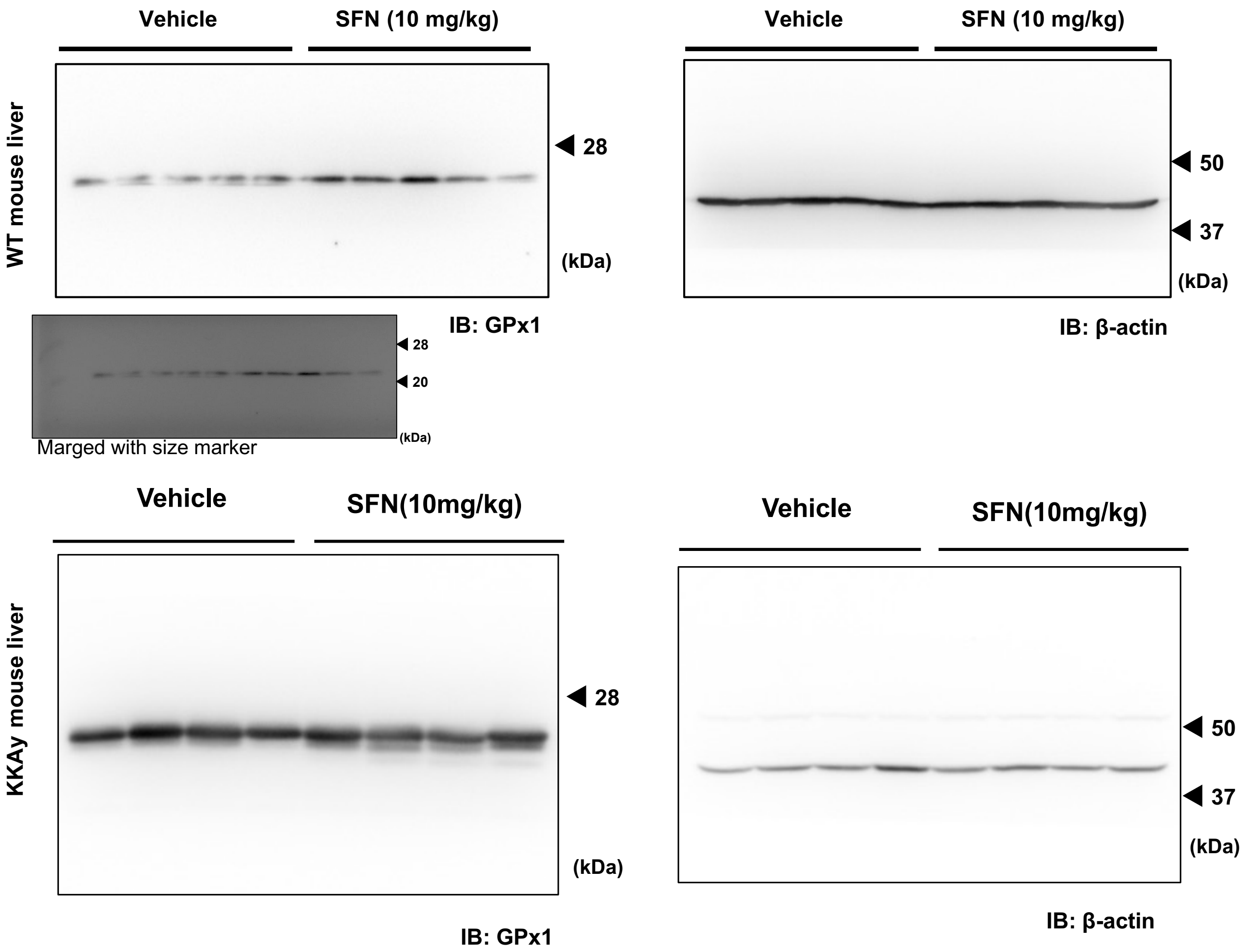

Supplemental Figure 17. Uncropped data of WB S7

b)

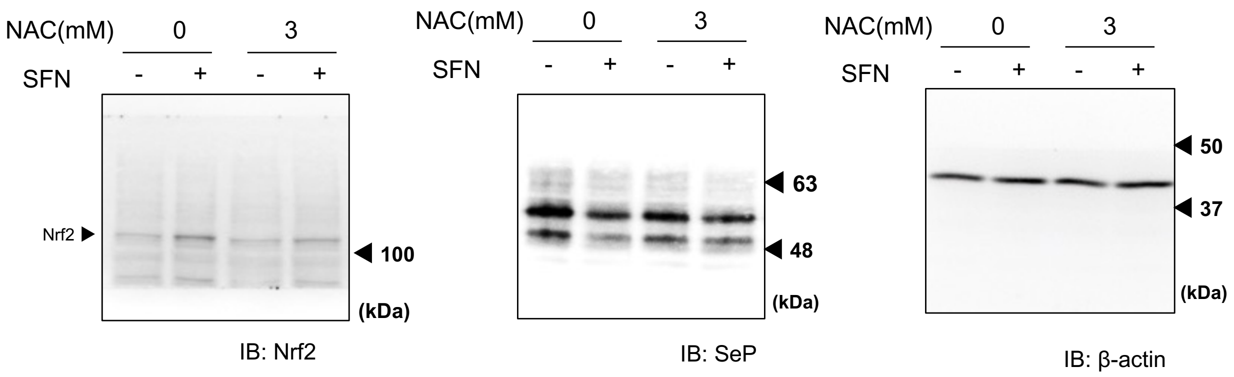

Supplemental Figure 18. Uncropped data of WB S9
