# Supplementary material for: Sulforaphane decreases serum selenoprotein P levels through enhancement of lysosomal degradation independent of Nrf2
Source: Commun Biol. 2023 Oct 19;6:1060. doi: 10.1038/s42003-023-05449-y (PMC10587141; doi:10.1038/s42003-023-05449-y)
Supplement: Supplementary file 4 — Reporting summary [file 42003_2023_5449_MOESM4_ESM.pdf]

nature portfolio

Corresponding author(s): Takashi Toyama

Last updated by author(s): Jul 21, 2023

Reporting Summary

Nature Portfolio wishes to improve the reproducibility of the work that we publish. This form provides structure for consistency and transparency in reporting. For further information on Nature Portfolio policies, see our Editorial Policies and the Editorial Policy Checklist.

Statistics

For all statistical analyses, confirm that the following items are present in the figure legend, table legend, main text, or Methods section.

n/a	Confirmed	
☐	☒	The exact sample size (n) for each experimental group/condition, given as a discrete number and unit of measurement
☐	☒	A statement on whether measurements were taken from distinct samples or whether the same sample was measured repeatedly
☐	☒	The statistical test(s) used AND whether they are one- or two-sided<br>*Only common tests should be described solely by name; describe more complex techniques in the Methods section.*
☐	☒	A description of all covariates tested
☐	☒	A description of any assumptions or corrections, such as tests of normality and adjustment for multiple comparisons
☐	☒	A full description of the statistical parameters including central tendency (e.g. means) or other basic estimates (e.g. regression coefficient) AND variation (e.g. standard deviation) or associated estimates of uncertainty (e.g. confidence intervals)
☒	☐	For null hypothesis testing, the test statistic (e.g. F, t, r) with confidence intervals, effect sizes, degrees of freedom and P value noted<br>*Give P values as exact values whenever suitable.*
☒	☐	For Bayesian analysis, information on the choice of priors and Markov chain Monte Carlo settings
☒	☐	For hierarchical and complex designs, identification of the appropriate level for tests and full reporting of outcomes
☒	☐	Estimates of effect sizes (e.g. Cohen's d, Pearson's r), indicating how they were calculated

Our web collection on statistics for biologists contains articles on many of the points above.

Software and code

Policy information about availability of computer code

Data collection	CFX Maestro (qPCR), ImageSaver6 (Western blottings), and fluoview (fluorescence images).
Data analysis	Graphpad Prism and ImageJ were used.

For manuscripts utilizing custom algorithms or software that are central to the research but not yet described in published literature, software must be made available to editors and reviewers. We strongly encourage code deposition in a community repository (e.g. GitHub). See the Nature Portfolio guidelines for submitting code & software for further information.

Data

Policy information about availability of data

All manuscripts must include a data availability statement. This statement should provide the following information, where applicable:

- Accession codes, unique identifiers, or web links for publicly available datasets
- A description of any restrictions on data availability
- For clinical datasets or third party data, please ensure that the statement adheres to our policy

The data supporting this manuscript are available from the corresponding author by reasonable request.

Research involving human participants, their data, or biological material

Policy information about studies with human participants or human data. See also policy information about sex, gender (identity/presentation), and sexual orientation and race, ethnicity and racism.

Reporting on sex and gender	No studies with human participants are included in the manuscript.
Reporting on race, ethnicity, or other socially relevant groupings	No studies with human participants are included in the manuscript.
Population characteristics	No studies with human participants are included in the manuscript.
Recruitment	No studies with human participants are included in the manuscript.
Ethics oversight	No studies with human participants are included in the manuscript.

Note that full information on the approval of the study protocol must also be provided in the manuscript.

Field-specific reporting

Please select the one below that is the best fit for your research. If you are not sure, read the appropriate sections before making your selection.

☒ Life sciences ☐ Behavioural & social sciences ☐ Ecological, evolutionary & environmental sciences

For a reference copy of the document with all sections, see nature.com/documents/nr-reporting-summary-flat.pdf

Life sciences study design

All studies must disclose on these points even when the disclosure is negative.

Sample size	No statistical method was used to determine sample size.
Data exclusions	No data was excluded
Replication	Biochemical experiments were examined at least three times independently, and their average or representative ones were shown in the manuscript
Randomization	No randomization was performed in this study
Blinding	No blinding was performed in this study

Reporting for specific materials, systems and methods

We require information from authors about some types of materials, experimental systems and methods used in many studies. Here, indicate whether each material, system or method listed is relevant to your study. If you are not sure if a list item applies to your research, read the appropriate section before selecting a response.

Materials & experimental systems

n/a	Involved in the study	
☐	☒	Antibodies
☐	☒	Eukaryotic cell lines
☒	☐	Palaeontology and archaeology
☐	☒	Animals and other organisms
☒	☐	Clinical data
☒	☐	Dual use research of concern
☒	☐	Plants

Methods

n/a	Involved in the study	
☒	☐	ChIP-seq
☒	☐	Flow cytometry
☒	☐	MRI-based neuroimaging

Antibodies

Antibodies used	anti-β-actin A5441, clone AC-15, anti-GPx1 ab108427, anti-GPx4 ab125066, anti-HO-1 sc-390991, anti-LAMP2 sc-18822, anti-LC-3A/B 12741S, anti-Nrf2 sc-365949, anti-p62 5114S and anti-hSeP & anti-mSeP were established at our previous report (Mita et al.,

nature portfolio | reporting summary

	2017). Detailed list of antibodies were shown in Supplementary information Table 1.
Validation	chrome-extension://efaidnbmnnnibpcajpcglclefindmkaj/https://www.sigmaaldrich.com/specification-sheets/141/510/A5441-BULK________SIGMA____.pdf<br>https://www.abcam.co.jp/products/primary-antibodies/glutathione-peroxidase-1-antibody-epr3312-ab108427.html<br>https://www.abcam.co.jp/products/primary-antibodies/glutathione-peroxidase-4-antibody-epncir144-ab125066.html<br>https://www.scbt.com/ja/p/heme-oxygenase-1-antibody-f-4<br>https://www.scbt.com/ja/p/lamp-2-antibody-h4b4<br>https://www.cellsignal.jp/products/primary-antibodies/lc3a-b-d3u4c-xp-rabbit-mab/12741<br>https://www.scbt.com/ja/p/nrf2-antibody-a-10<br>https://www.cellsignal.jp/products/primary-antibodies/sqstm1-p62-antibody/5114<br><br>Selenoprotein P antibodies were validated using serum of SeP KO mice in this study and previous report (Mita et al., 2017).

Eukaryotic cell lines

Policy information about cell lines and Sex and Gender in Research

Cell line source(s)	HepG2 (derived from human liver carcinoma, which obtained from JCRB cell bank)
Authentication	No further authentication was performed for the cell line
Mycoplasma contamination	Not tested for mycoplasma contamination
Commonly misidentified lines (See ICLAC register)	No commonly misidentified lines were used

Animals and other research organisms

Policy information about studies involving animals; ARRIVE guidelines recommended for reporting animal research, and Sex and Gender in Research

Laboratory animals	male 6-week-old C57BL/6J mice (Claire Japan), Male 16-week-old KKAy mice (CLEA Japan, Kanagawa, Japan), and male 6-week-old Nrf2 (-/-) mice, Keap1flox/flox::Alb-Cre mice, and Selenoprotein P (-/-) mice were used in this study.
Wild animals	No wild animals are used in this study
Reporting on sex	In this study, experiments were conducted using only males, and differences between males and females were not taken into account.
Field-collected samples	No field-collected samples were used
Ethics oversight	The animal study was carried out in accordance with the rules and guidelines for the proper implementation of animal experiments at Tohoku University (Approval No. 2019-018-05)

Note that full information on the approval of the study protocol must also be provided in the manuscript.
